# Electron-Deficient Acetylenes as Three-Modal Adjuvants in S_N_^H^ Reaction of Pyridinoids with Phosphorus Nucleophiles

**DOI:** 10.3390/molecules26226824

**Published:** 2021-11-11

**Authors:** Boris A. Trofimov, Pavel A. Volkov, Anton A. Telezhkin

**Affiliations:** A.E. Favorsky Irkutsk Institute of Chemistry, Siberian Branch, Russian Academy of Sciences, 1 Favorsky Str., 664033 Irkutsk, Russia; volkov_p_a@irioch.irk.ru (P.A.V.); telezhkin@irioch.irk.ru (A.A.T.)

**Keywords:** electron-deficient acetylenes, PH–nucleophiles, pyridines, quinolines, isoquinolines, acridine, S_N_^H^ reaction

## Abstract

Publications covering a new easy metal-free functionalization of pyridinoids (pyridines, quinolines, isoquinolines, acridine) under the action of the system of electron-deficient acetylenes (acetylenecarboxylic acid esters, acylacetylenes)/*P*-nucleophiles (phosphine chalcogenides, *H*-phosphonates) are reviewed. Special attention is focused on a S_N_^H^ reaction of the regioselective cross-coupling of pyridines with secondary phosphine chalcogenides triggered by acylacetylenes to give 4-chalcogenophosphorylpyridines. In these processes, acetylenes act as three-modal adjuvants (i) activating the pyridine ring towards *P*-nucleophiles, (ii) deprotonating the P-H bond and (iii) facilitating the nucleophilic addition of the *P*-centered anion to a heterocyclic moiety followed by the release of the selectively reduced acetylenes (*E*-alkenes).

## 1. Introduction

Phosphorylated heterocyclic compounds have attracted steadily growing attention as perspective pharmaceuticals or their precursors [1,2,3], multipurpose ligands for the design of biologically active metal complexes [4,5,6,7] and catalysts [8,9,10,11], as well as building blocks for organic synthesis [12,13,14,15]. Among them, pyridines and their condensed derivatives (especially quinolines), with phosphorus-containing functional groups, are of particular research interest [1,2,5,6,7,8,9,10,11,12,13,14].

Significant endeavors have been directed towards the synthesis of such compounds, a transition metal-free nucleophilic substitution of hydrogen in a heteroaromatic ring (S_N_^H^ reaction) by phosphorus-centered nucleophiles being one of the most dynamically developing approaches because of its apparent benefits (environmental friendliness, no pre-functionalization of the starting materials, no toxic waste). This has been evidenced by a series of reviews [16,17,18,19,20,21,22,23,24] by Mąkosza’s [16,22,25,26,27,28] and Chupakhin’s [18,19,20,21,23,24,29,30,31,32] groups, who pioneered this chemistry. Commonly, these processes meet three requirements: (i) the electrophilic activation of the aromatic ring, (ii) generation of the attacking anion with strong bases and (iii) aromatization of anionic σ_H_-adducts or dihydro intermediates by external oxidants. As a rule, the nitro-substituents, or N-O bonds (*N*-oxide moiety), are employed activating groups. In particular, this was shown by the phosphorylation of nitroarene with phosphine anions, followed by the permanganate oxidation of the anionic species [27,28] or by the phosphorylation of acridinium salts with trialkylphosphite [32,33,34]. The extrinsic activation of quinolines, phenanthrolines and naphthyridines by sulfuric acid towards a nucleophilic attack by dimethyltrimethylsilylphosphite leading to the double phosphorylation of the heterocycle has also been implemented [35,36].

A short time ago [37,38,39,40], the nucleophilic substitution of hydrogen (S_N_^H^ reaction) in non-activated pyridines and their fused derivatives by secondary phosphine chalcogenides (synthesized from red phosphorus [41,42,43,44]) in the presence of electron-deficient terminal and internal acetylenes was disclosed. These reactions involve the step of *N*-vinylation/*C*-phosphorylation of pyridinoids, and are often retained at this step [45,46,47,48,49]. Afterwards, a series of this finding’s follow-up works appeared.

This review covers publications (since 1999) concerning the reactions of pyridinoids with PH–nucleophiles in the presence of electron-deficient acetylenes which act as trimodal (polarization/deprotonation/oxidation) catalyst-like adjuvants.

## 2. S_N_^H^ Phosphorylation of Pyridines Triggered and Driven by Electrophilic Acetylenes

### 2.1. One-Step S_N_^H^ Phosphorylation

**Scheme 1:**
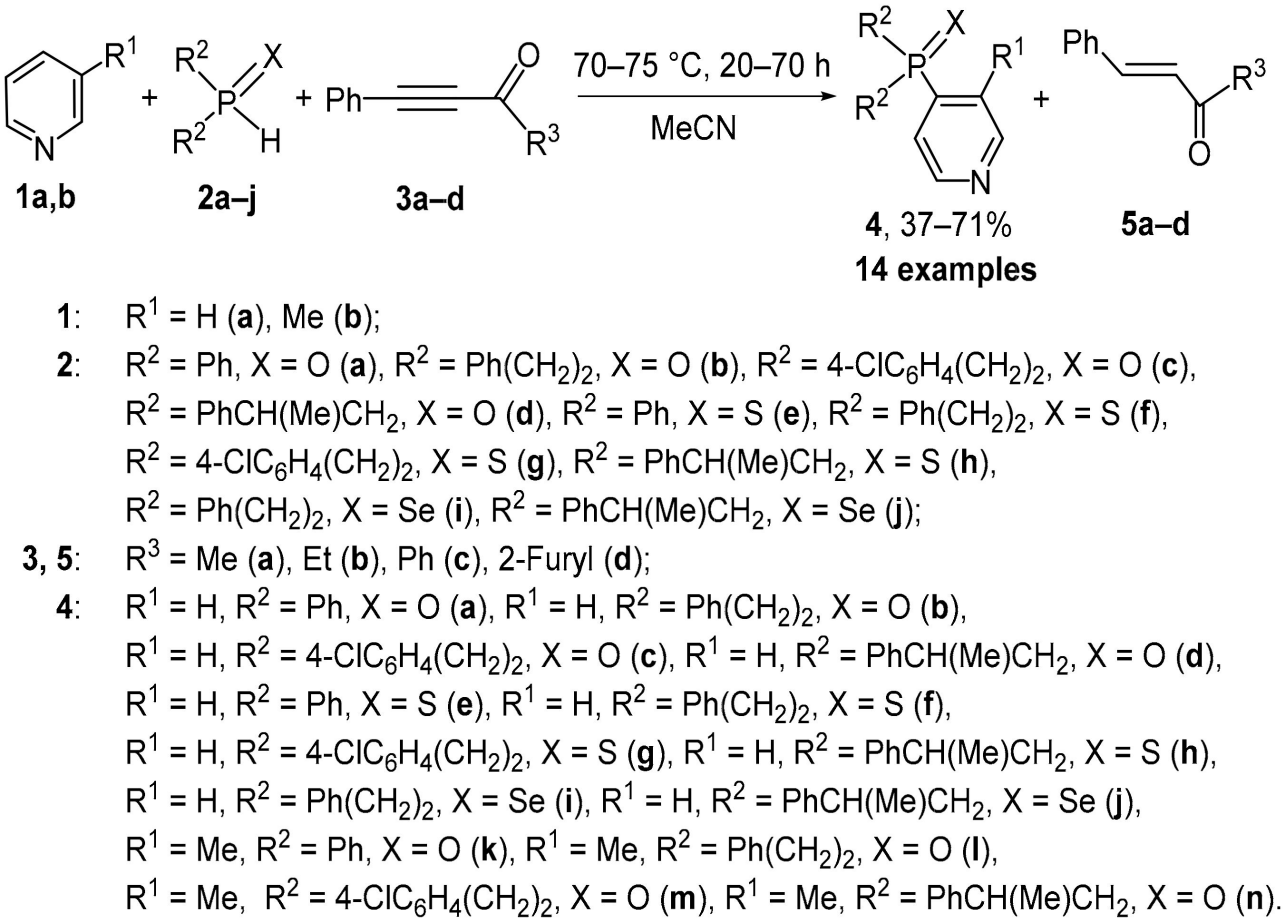
S_N_^H^ cross-coupling of pyridines with secondary phosphine chalcogenides in the presence of acylphenylacetylenes.

In 2018, it was found that the regioselective oxidative cross-coupling reaction between pyridines **1a**,**b** and secondary phosphine chalcogenides **2a–j** was triggered and further driven by electrophilic acetylenes **3a–d** to produce S_N_^H^ products, phosphorylated pyridines **4a–n**, and *E*-acylphenylethenes **5a–d** (Scheme 1) [37]. The reaction tolerates a quite representative number of structurally diverse secondary phosphine chalcogenides (oxides, sulfides and selenides), having both aromatic and alkyl aromatic substituents.

**Scheme 2:**
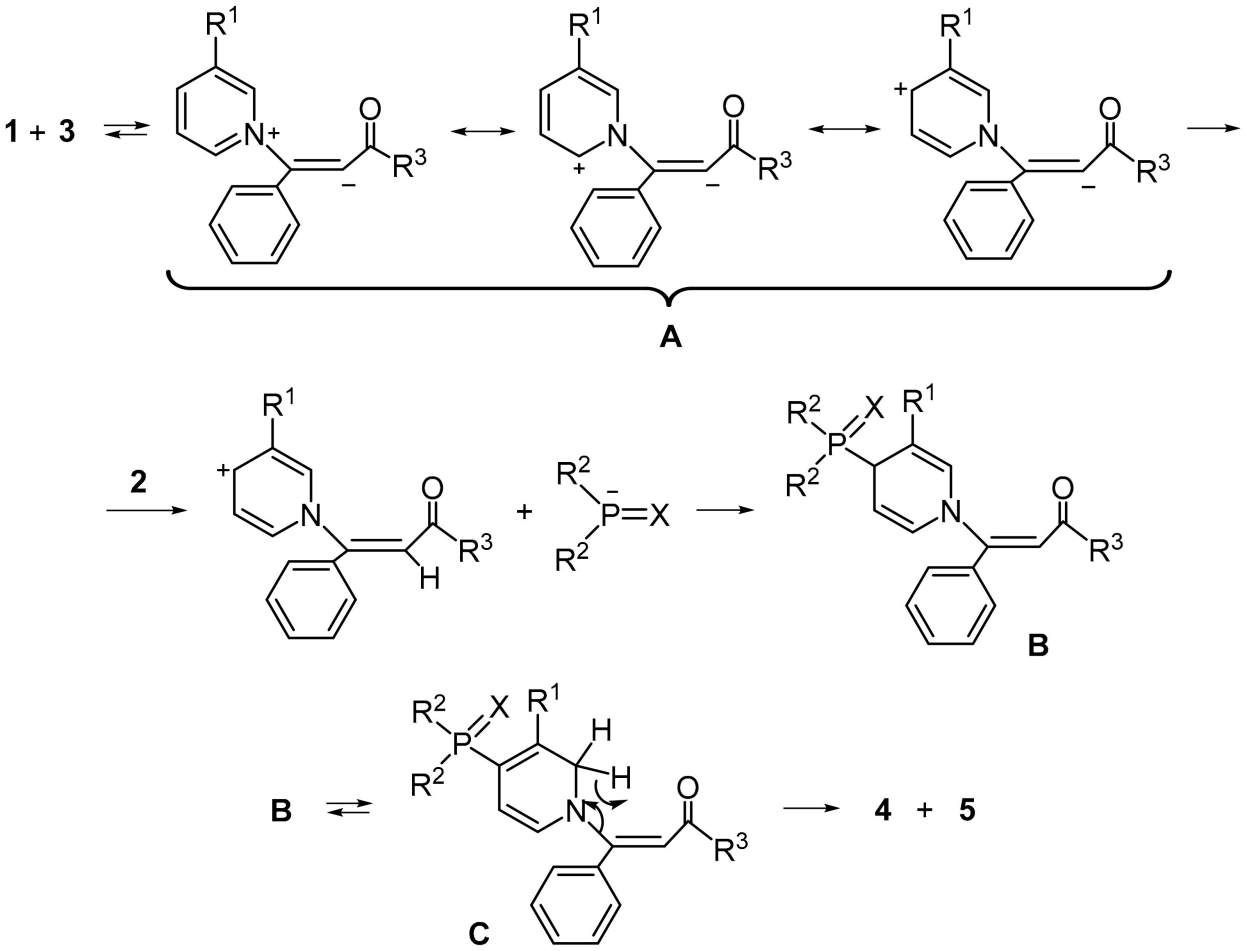
A plausible reaction pathway.

The reaction involves the reversible generation of 1,3-dipole **A** via a nucleophilic attack of pyridine nitrogen at the electron-deficient triple bond (Scheme 2) [37].

The intermediate **A** accepts a proton from the phosphine chalcogenides **2** to produce the *P*-centered anion, which regioselectively attacks position four of the pyridine ring delivering dihydropyridine **B**. Its aromatization occurs via the stereoselective elimination of *E*-acylphenylethenes **5** from isomerized intermediate **B⇌C**. The elimination includes the transfer of the hydride anion from position two of dihydropyridine to emerging carbocation. The driving force of the elimination is likely a higher thermodynamic stability of the final products (phosphorylated pyridines **4** and conjugated functionalized ethenes **5**) as compared to intermediates **B⇌C**.

**Scheme 3:**
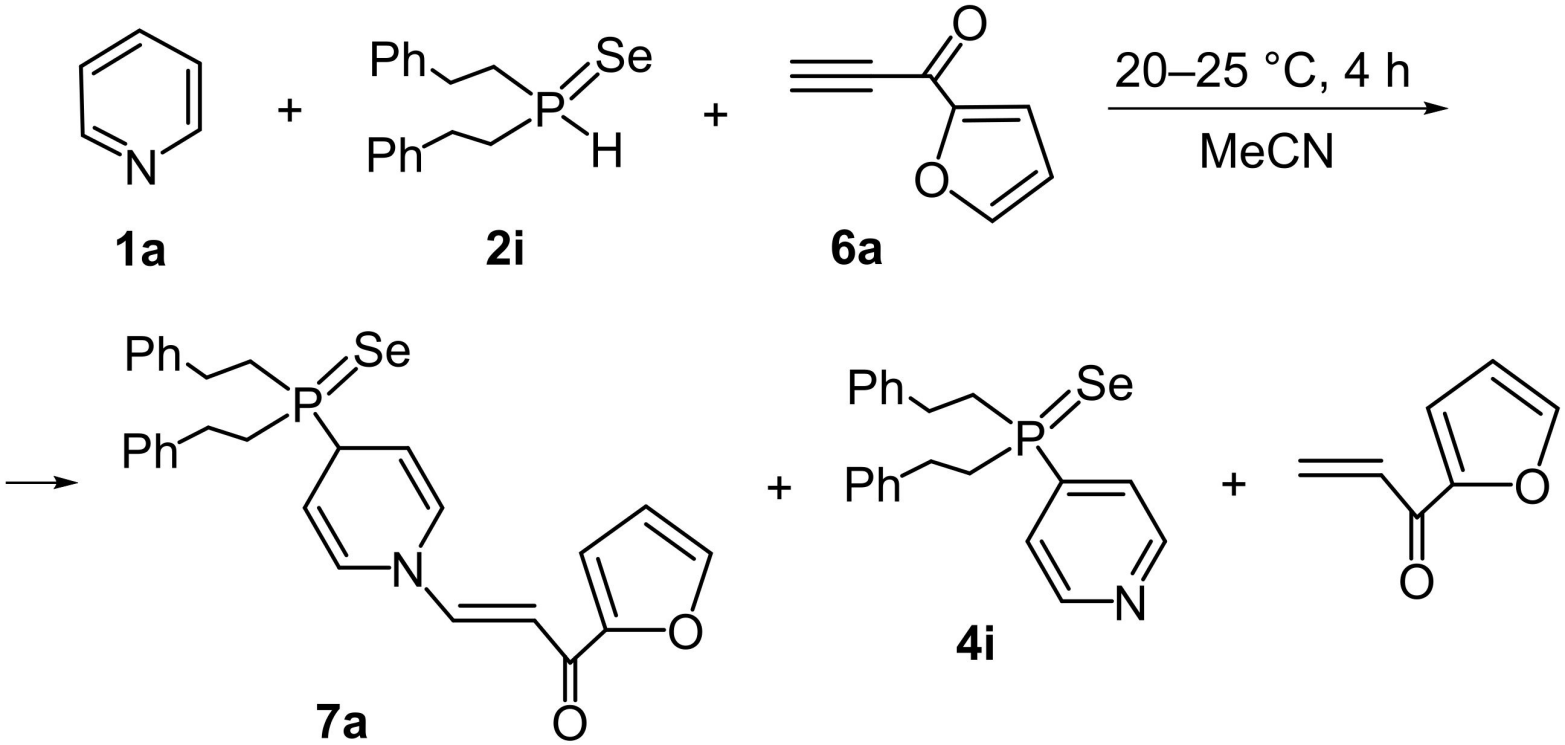
Reaction of pyridine, phosphine selenide and furoylacetylene.

The mechanism is supported by the detection of the intermediate dihydropyridine **7a** in a mixture (^1^H and ^31^P NMR) with the corresponding pyridine **4i**, when pyridine **1a** reacts (room temperature, MeCN, 4 h) with terminal furoylacetylene **6a** (having no phenyl substituent in position two) and phosphine selenide **2i** (Scheme 3).

The perdeuteropyridine (pyridine-*d_5_*) with phosphine sulfide **2f** and acetylene **3c** afforded the isotopically labeled cross-coupling product, 4-[bis(2-phenylethyl)thiophosphoryl]pyridine-*d_4_*, in a 42% yield (for 50 h) [37]. The low yield and longer reaction time compared to those for non-deuterated pyridine (71%, 35 h) are likely due to the deuterium kinetic isotopic effect. Notably, 3-fluoropyridine appeared unable to undergo above cross-coupling, obviously because of its lower basicity/nucleophilicity (an electron-acceptor effect of the fluorine atom).

**Scheme 4:**
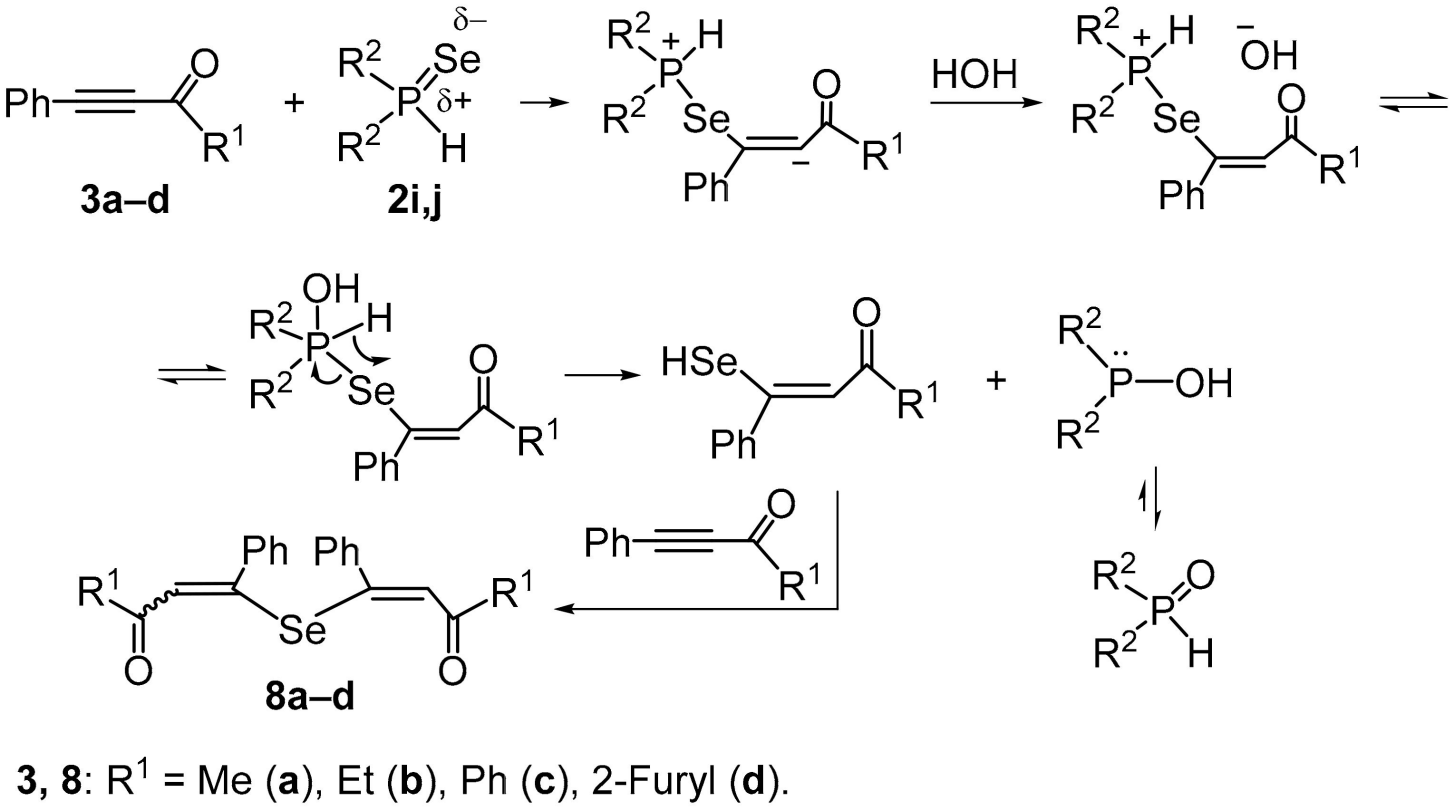
Selenylation of the acylphenylacetylenes by secondary phosphine selenides.

The observed moderate yields of the cross-coupling products with phosphine selenides **2i,j** (37–40%) vs. the ones with phosphine oxides and sulfides [37] (55–71%) are due to the side selenylation of the starting acetylenes **3** to divinyl selenides **8** (Scheme 4).

The unusual selenium transfer from secondary phosphine selenides **2i,j** to the electron-deficient triple bond involves trace water and can also proceed in aqueous media (70–72 °C, 3 h) [50,51].

**Scheme 5:**
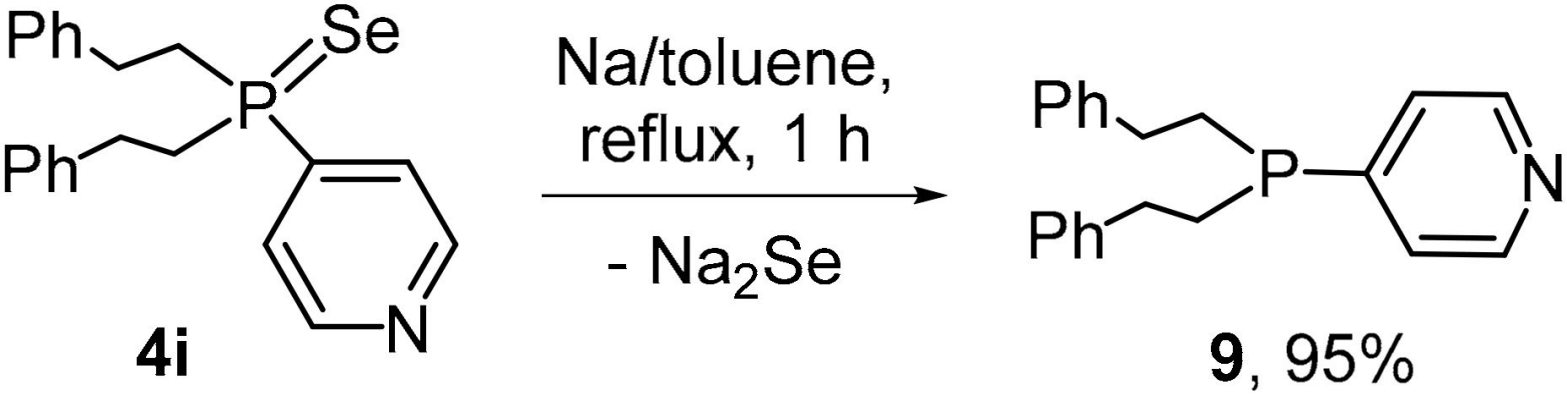
Synthesis of 4-pyridyl-bis(2-phenylethyl)phosphine.

The above cross-coupling might open simple access to the related phosphines, sought-after ligands for new catalytically active metal complexes, having been demonstrated by the reduction in selenophosphorylpyridine **4i** to phosphine **9** (Scheme 5) [37].

The improved green route towards 4-chalcogenophosphorylpyridines **4** was also developed [38]. Therefore, it was found that the previously considered S_N_^H^ cross-coupling of pyridine **1a** and 3-methylpyridine **1b** with bis(2-phenylethyl)phosphine oxide **2b** or bis(2-phenylethyl)phosphine sulfide **2f** in the presence of benzoylphenylacetylene **3c** as an adjuvant can be realized in a solvent-free version for a much shorter time (5.5–7 h), similar to Scheme 1. This greener protocol for the synthesis of phosphorylated pyridines **4** is not only environmentally friendly, but in some cases provides better average yields.

**Scheme 6:**
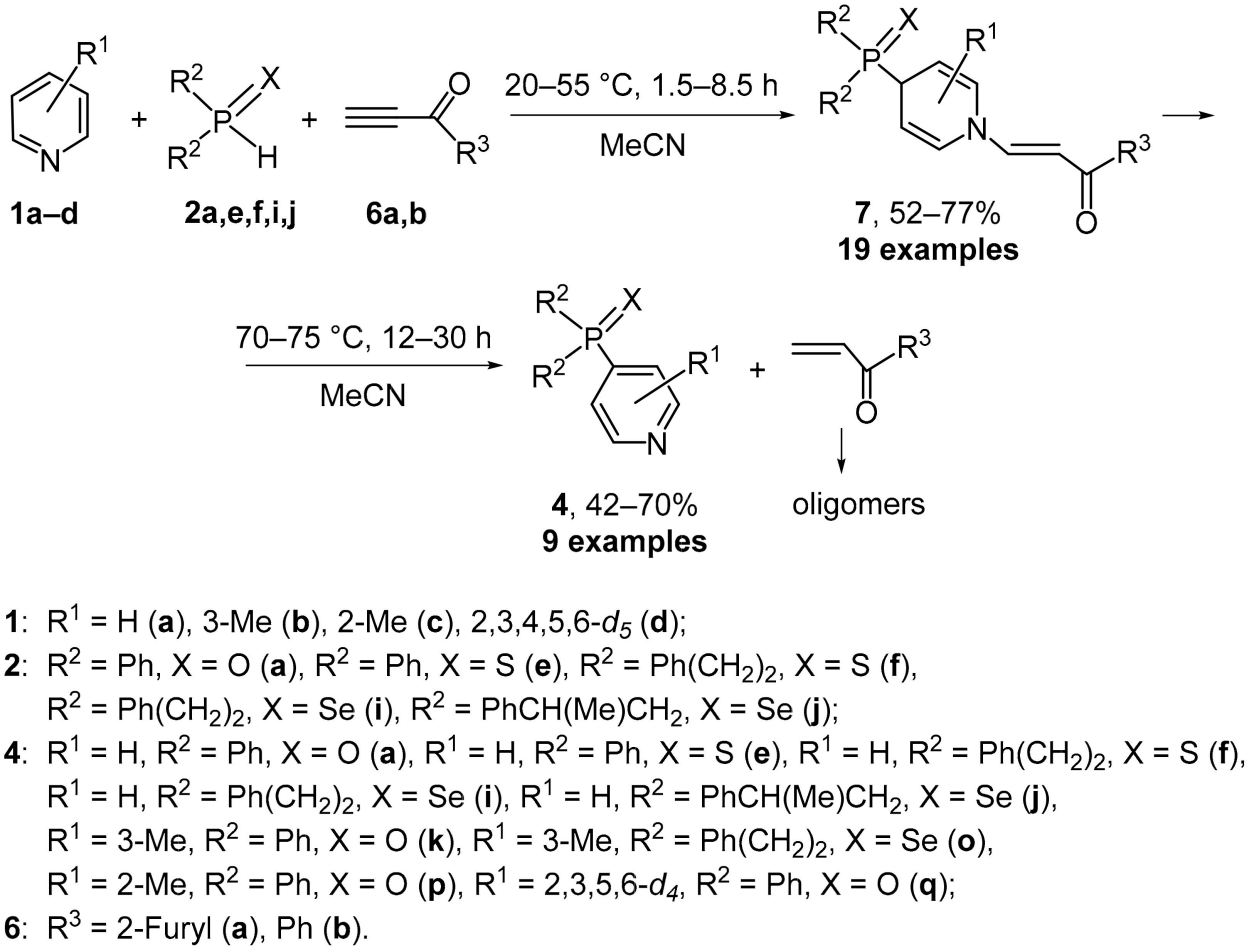
Formation and aromatization of dihydropyridines via elimination of vinyl ketones.

Terminal acylacetylenes **6a,b** (2-furoyl- and benzoylacetylene) can also behave as adjuvants triggering and driving S_N_^H^ cross-coupling of pyridines **1** with secondary phosphine chalcogenides **2** under similar conditions to afford 4-chalcogenophosphorylpyridines **4** in up to a 70% yield (Scheme 6). In the reaction, intermediate 1-acylvinyl-4-chalcogenophosphoryl dihydropyridines **7** were isolated (in 52–77% yields, see also Scheme 21 below), further undergoing the oxidative (relative to the dihydropyridine ring) elimination of vinyl ketone oligomers [39].

**Scheme 7:**
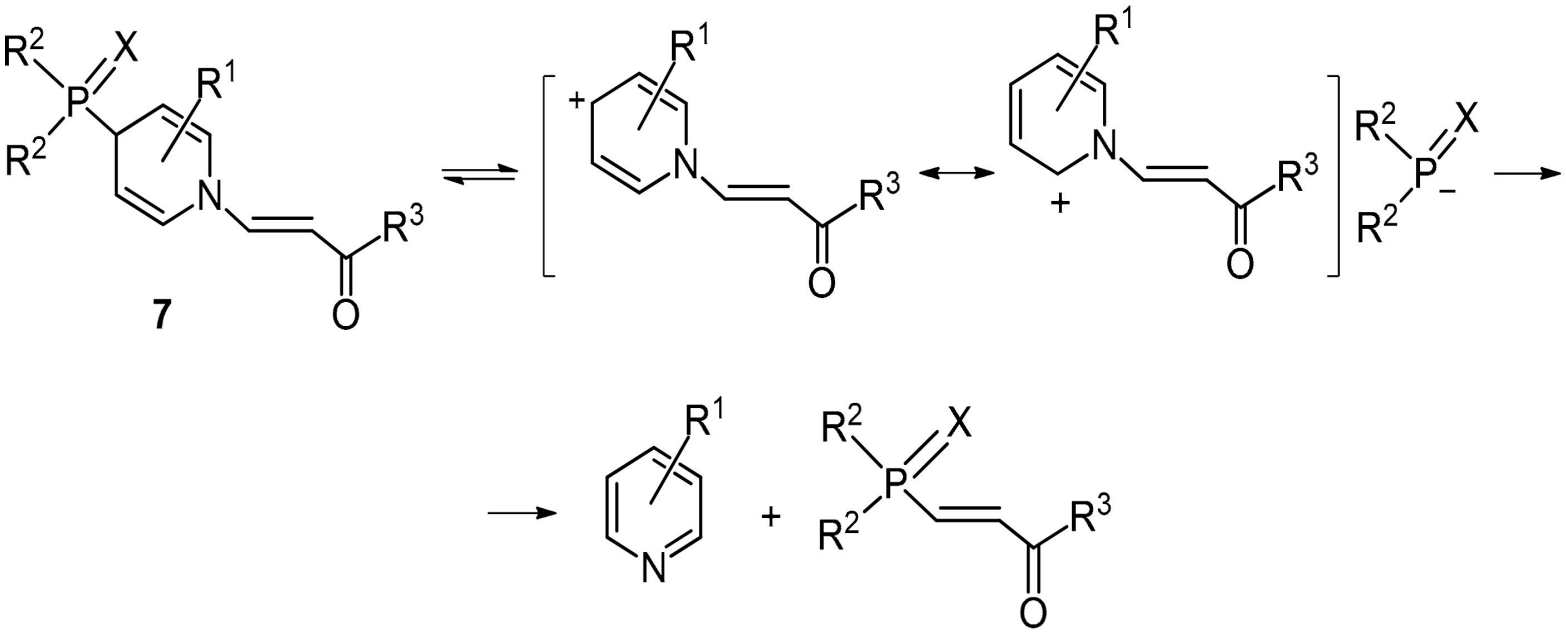
Plausible retro-aromatization of dihydropyridines.

The cross-coupling starts with the prototropic shift in the 1,4-dihydropyridine ring to 1,2-isomers, from which the redox elimination of acylethenes takes place easier as a concerted step process. The moderate yields of the target products **4** and the presence of acylphosphorylethenes of phosphine chalcogenides to acylacetylenes in the reaction mixture were assigned [39] to the retro-aromatization of intermediates **7** to the starting pyridines. This likely resulted from the dissociation of the P-C bond and recombination of chalcogenophosphoryl anions with the leaving acylvinyl cations (Scheme 7).

**Scheme 8:**
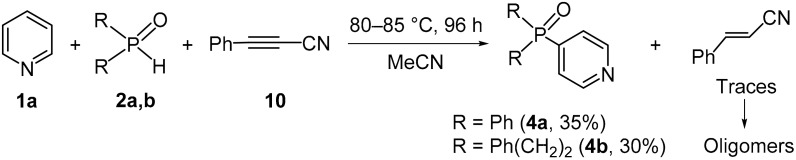
S_N_^H^ cross-coupling between pyridine and secondary phosphine oxides in the presence of 3-phenyl-2-propynenitrile.

The reflux (80–85 °C, 96 h) of pyridine **1a**, 3-phenyl-2-propynenitrile **10** and phosphine oxides **2a,b** in MeCN led to the regioselective phosphorylation of pyridine to 4-phosphorylpyridines **4a,b** in a 30–35% yield and 3-phenyl-2-propenenitrile oligomers (Scheme 8) [40].

All the above information has allowed for the generalization of a concept of trimodal (polarization/deprotonation/oxidation) adjuvant-like assistance of electron-deficient acetylenes in the S_N_^H^ reaction of the pyridinoid heterocycles with PH–nucleophiles. This includes: (i) the repolarization of the pyridine ring, (ii) generation of phosphorus-centered anions by the deprotonation of the P-H bond and (iii) oxidation of the dihydropyridine ring.

### 2.2. N-Vinylated/C-Phosphorylated Pyridines: Delayed S_N_^H^ Phosphorylation

As follows from the preceding Section, the possibility to stop the S_N_^H^ phosphorylation of pyridines with secondary phosphine chalcogenides in the presence of electron-deficient acetylenes as adjuvants on the intermediate step, i.e., the formation of *N*-vinylated/*C*(4)-phosphorylated 1,4-dihydropyridines, provided a novel efficient approach to the synthesis of earlier inaccessible richly functionalized dihydropyridines.

**Scheme 9:**
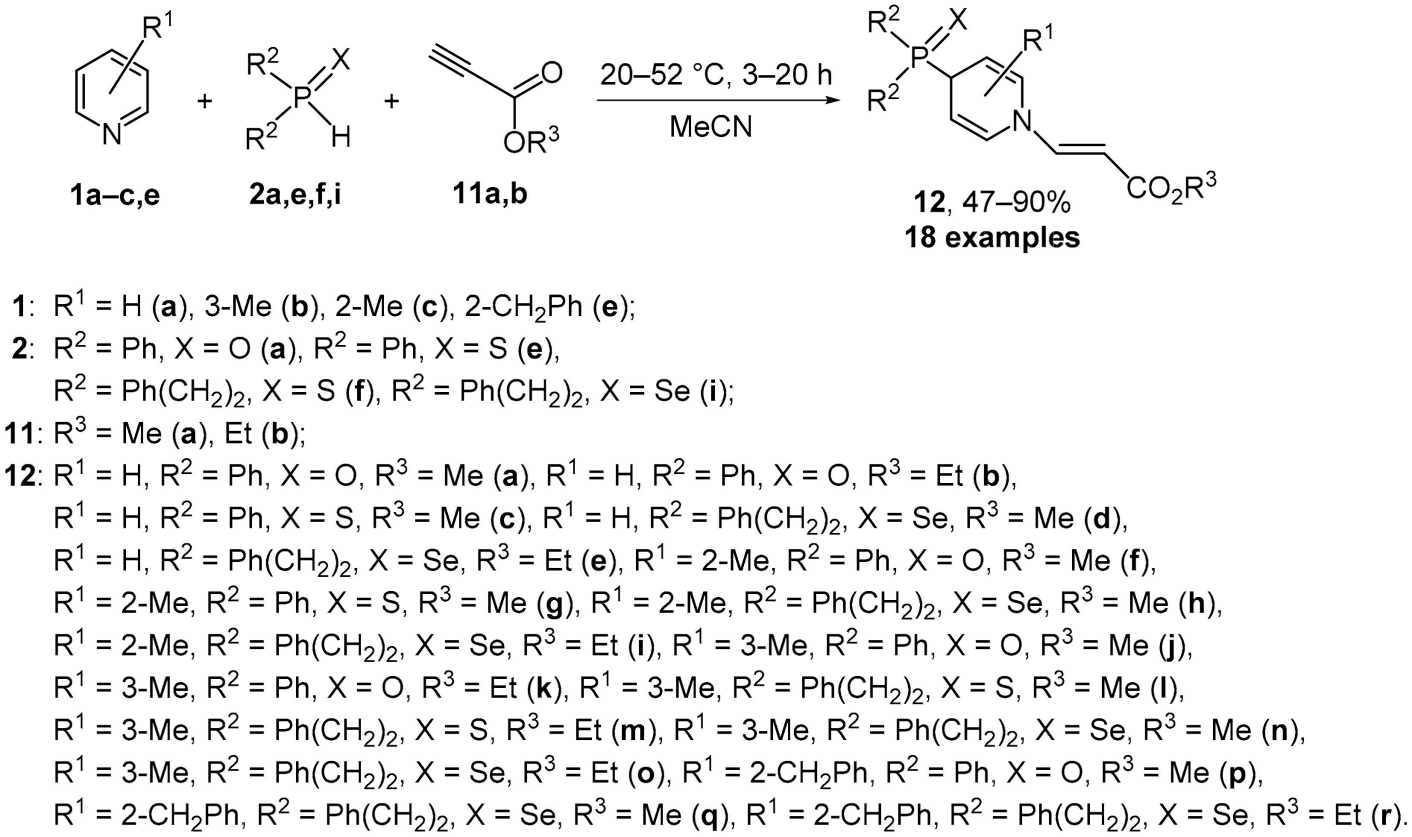
Reductive *N*-vinylation/*C*-phosphorylation of pyridines with alkyl propiolates and secondary phosphine chalcogenides.

The synthesis of *N*-vinylated/*C*(4)-phosphorylated 1,4-dihydropyridines **12a–r** in a 47–90% yield from pyridines **1**, alkyl propiolates **11** and secondary phosphine chalcogenides **2** was implemented as a one-pot three-component reaction at 20–52 °C for 3–20 h (Scheme 9) [45,47].

The reactions involving pyridine **1a** and secondary phosphine sulfide **2f** or selenide **2i** proceeded at room temperature, though phosphine oxide **2a** required slight heating (50–52 °C), likely due to its lower acidity. The same was observed in the case of 2-methylpyridine **1c,** which we ascribed to steric hindrance.

The process is regioselective: the expected 1,2-dihydropyridines were not detected in the reaction mixture (^1^H, ^31^P NMR), though it was reported [52,53] that dialkyl (diphenyl) *H*-phosphonates reacted with electron-deficient acetylenes and pyridine to exclusively yield the corresponding *N*-ethenyl-1,2-dihydropyridines with (RO)_2_P(O)-group. The high stereoselectivity of the reaction (Scheme 9) is its advantage; the ethenyl moieties of 1,4-dihydropyridines **12** are of *E*-configuration exclusively.

**Scheme 10:**
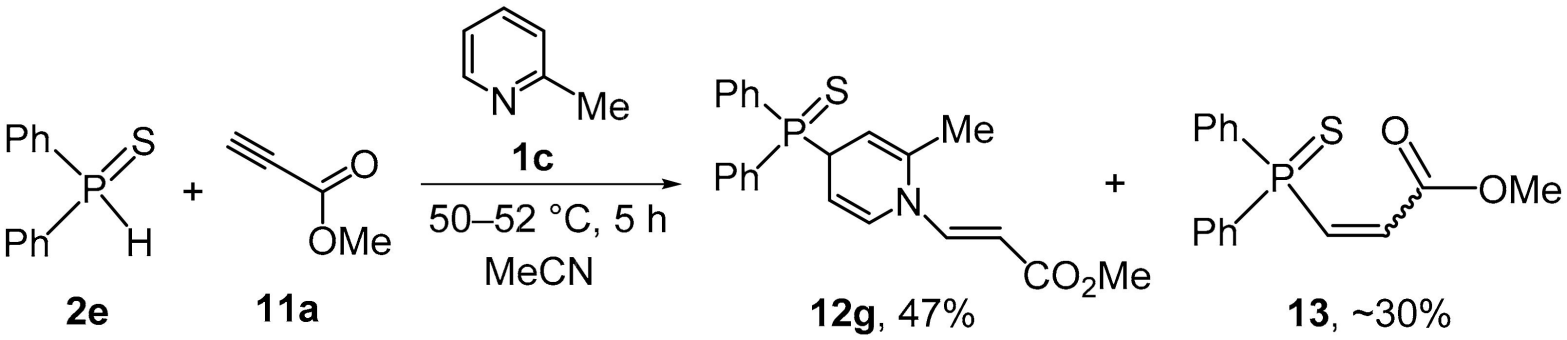
Side formation of diphenylphosphine sulfide to methyl propiolate adduct.

2-Methylpyridine **1c** with methyl propiolate **11a** and diphenylphosphine sulfide **2e** gave dihydropyridine **12g** in a 47% yield, along with *E-* and *Z*-isomers of diphenyl(methylpropenoate)phosphine sulfide **13** (content in the reaction mixture ≈30%, identified by ^31^P NMR) (Scheme 10) [45]. The side formation of vinylphosphine sulfide **13** was expected from the known data that secondary phosphine chalcogenides added to acyl- and cyanoacetylenes in the presence of the base [54].

**Scheme 11:**
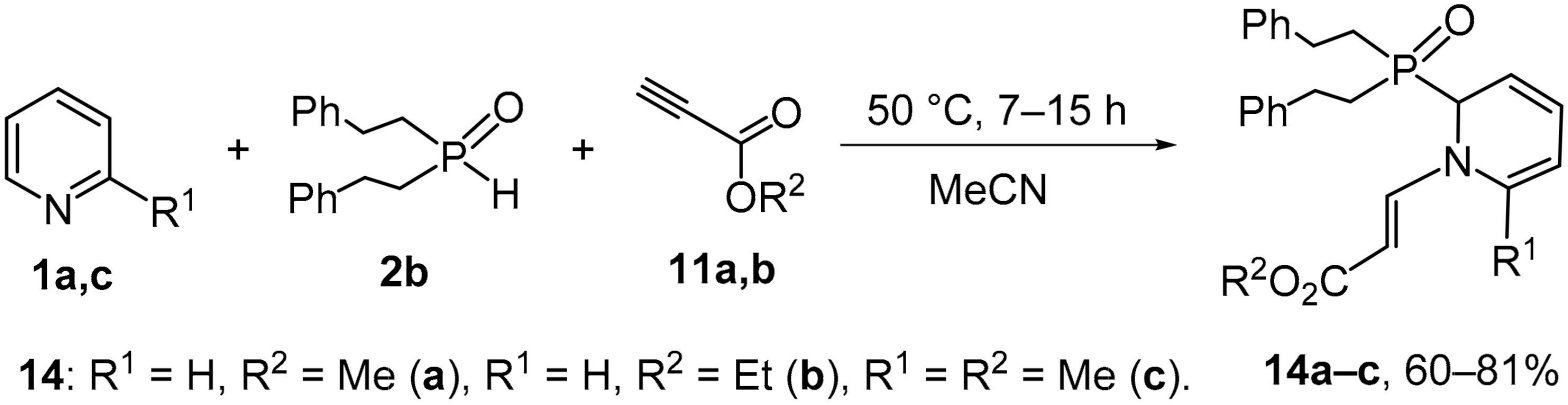
Reaction of pyridines with alkyl propiolates and bis(2-phenylethyl)phosphine oxide.

The replacement of diphenylphosphine oxide **2a** in this reaction by bis(2-phenylethyl)phosphine oxide **2b** changed the *C*-phosphorylation direction to the formation of 1-(*E*)-ethenyl-1,2-dihydropyridines **14a**–**c** in a 60–81% yield (Scheme 11) [46].

**Scheme 12:**
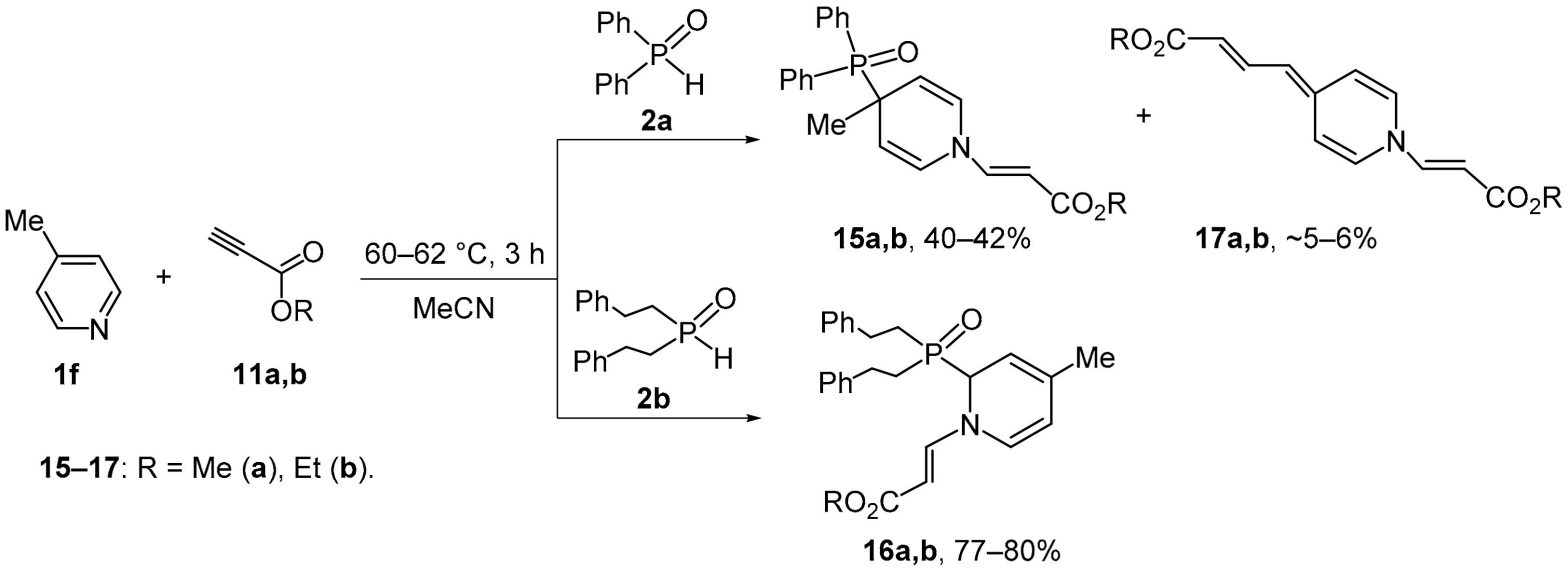
*N*-vinylation/*C*-phosphorylation of 4-methylpyridine with alkyl propiolates and secondary phosphine oxides.

4-Methylpyridine **1f** with alkyl propiolates **11a,b** and secondary phosphine oxides **2a**,**b** (60–62 °C, 3 h, MeCN) regio- and stereoselectively afforded 1-(*E*)-ethenyl-4-chalcogenophosphoryl-1,4-dihydropyridines **15a,b** (yield 40–42%) in the case of phosphine oxide **2a** or 1-(*E*)-ethenyl-2-chalcogenophosphoryl-1,2-dihydropyridines **16a,b** (yield 77–80%) when using phosphine oxide **2b**. Moderate yields of 1,4-dihydropyridines **15a,b** probably owe to the formation of side products **17a,b**, adducts of 4-methylpyridine to alkyl propiolates (Scheme 12) [48].

**Scheme 13:**
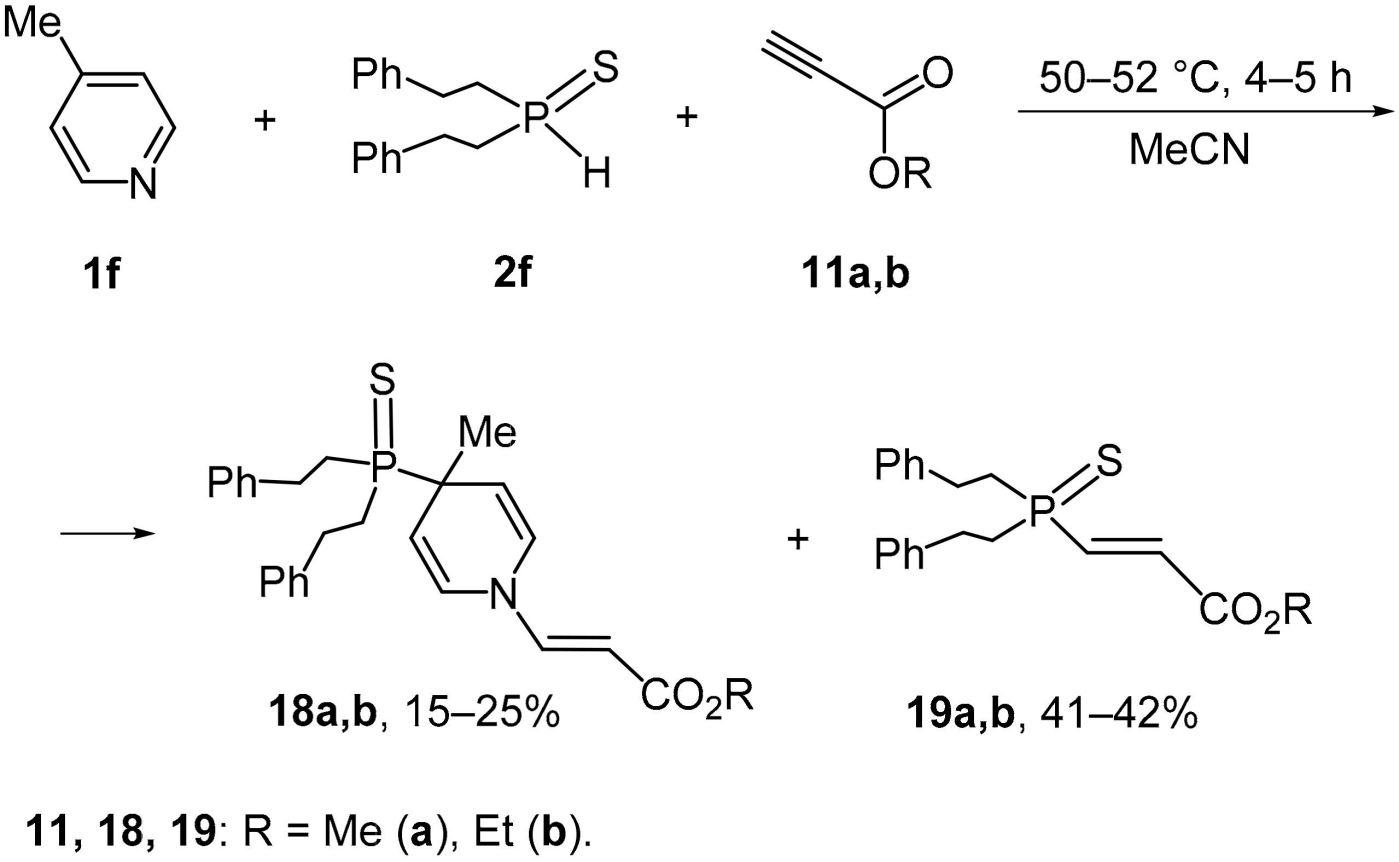
Side formation of bis(2-phenylethyl)phosphine sulfide to alkyl propiolates adducts.

Under similar conditions (50–52 °C, 4–5 h, MeCN), the three-component vinylation/phosphorylation reaction of 4-methylpyridine **1f** with secondary phosphine sulfide **2f** and propiolates **11a,b** was accompanied by the nucleophilic monoaddition of this PH–compound to the triple bond. As a result, 1-(*E*)-ethenyl-4-thiophosphoryl-1,4-dihydropyridines **18a,b** and the adducts **19a,b** were synthesized in 15–25% and 41–42% yields, respectively (Scheme 13) [47,48].

**Scheme 14:**
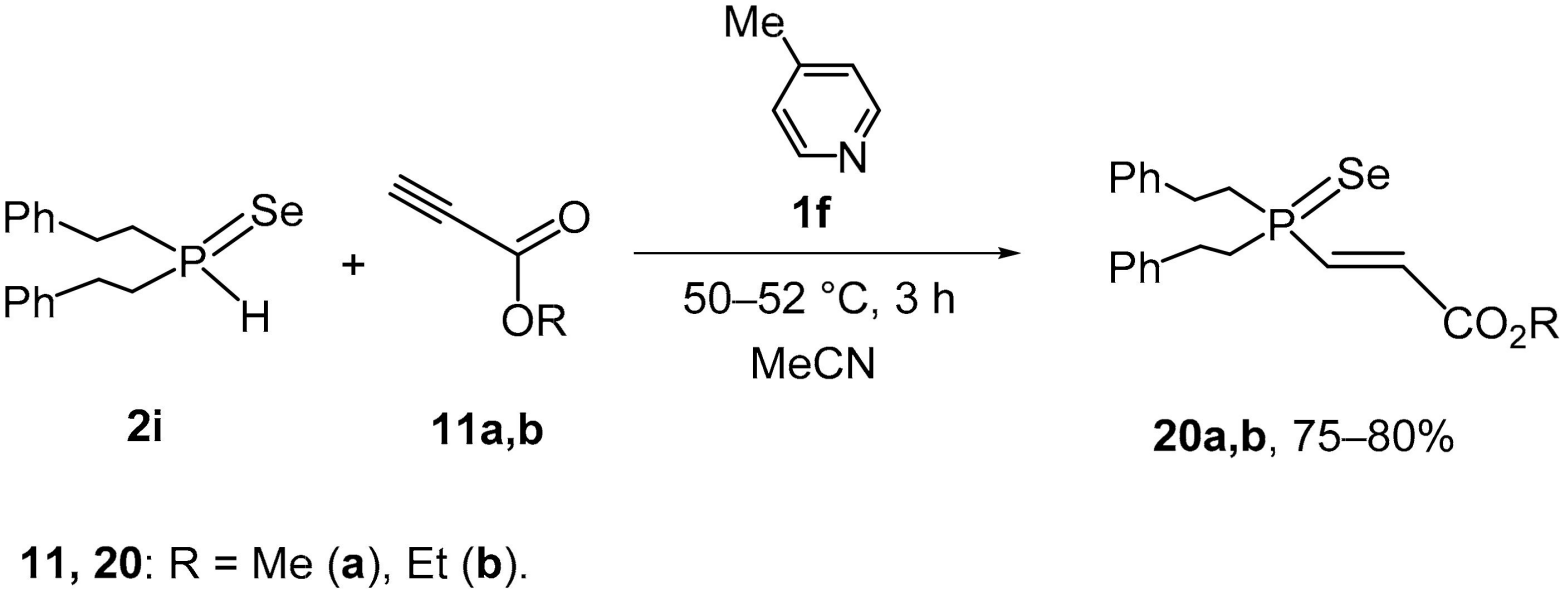
Formation of bis(2-phenylethyl)phosphine selenide to alkyl propiolate adducts.

Secondary phosphine selenide **2i** was not phosphorylated with 4-methylpyridine **1f** in the presence of propiolates **11a,b,** but instead formed adducts **20a,b** in a 75–80% yield (Scheme 14) [47,48].

**Scheme 15:**
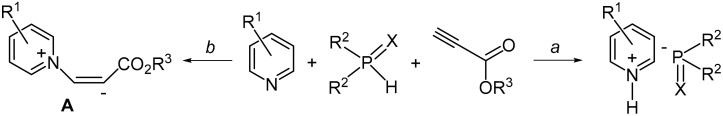
Protonation of pyridines (path *a*) and generation of zwitterions (path *b*).

To rationalize the formation of monoadducts **13**, **19** and **20,** especially with 4-methylpyridine (Scheme 12 and Scheme 13), the two competitive initial reactions should be analyzed [47]; the protonation of pyridines by the P-H bond (Scheme 15, *a*) and nucleophilic attack of pyridine nitrogen to the triple bond of alkyl propiolates to generate zwitterions **A** (Scheme 15, *b*).

Assumingly, the protonation of 4-methylpyridine as more basic than 2- and 3-methyl congeners (p*K*_a_ 6.05, 5.96, 5.68, respectively) is preferred, particularly by the most acidic secondary phosphine selenide **2i** (compared with other phosphine chalcogenides [55]). This is why a two-component interaction between phosphine chalcogenides and acetylenes **11** took over. 4-Methylpyridine, in this case, plays a role of a basic catalyst.

At the same time, because of the weaker acidity of the secondary phosphine oxide, zwitterion **A** is not neutralized with the proton (the phosphine oxide remaining intact), but further reacts with the second molecule of methyl propiolate as an electrophile to furnish the Acheson adduct [48].

**Scheme 16:**
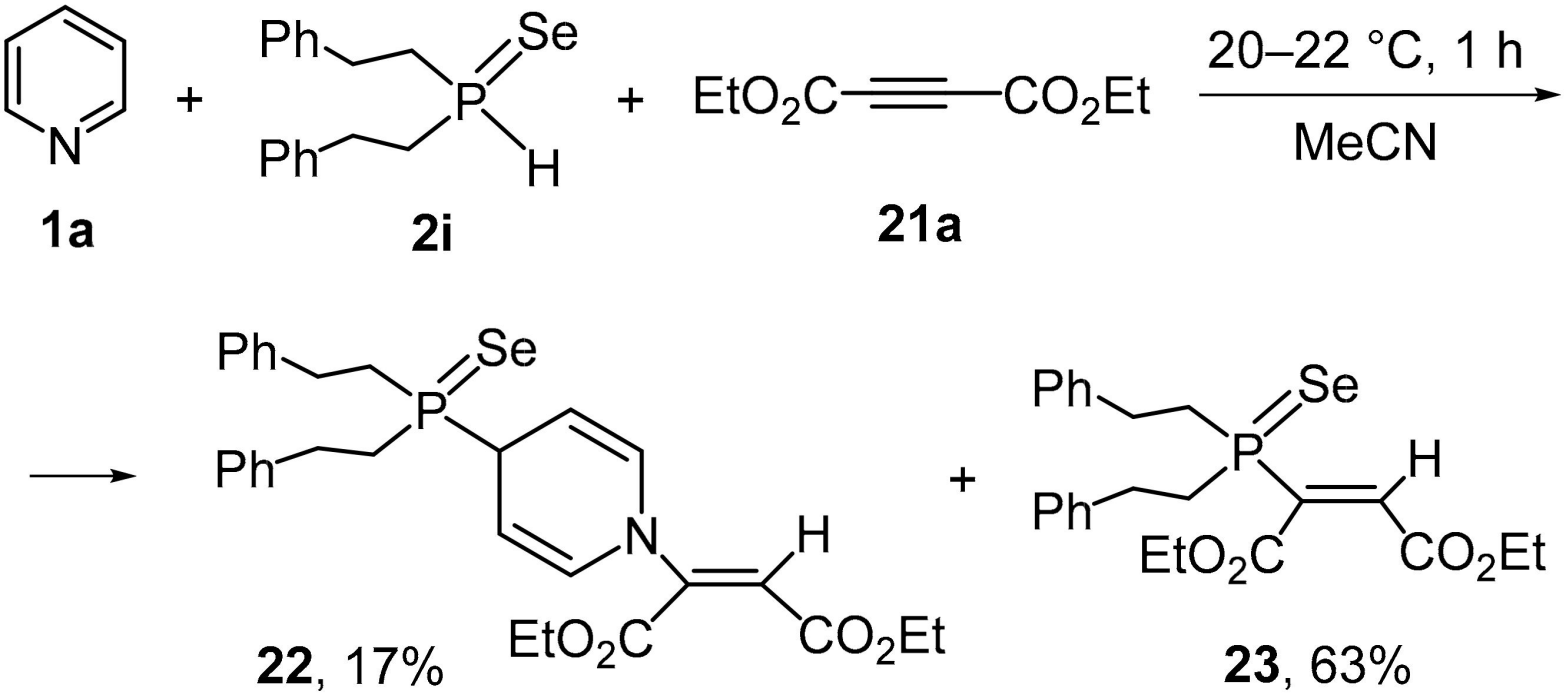
Reaction of pyridine, diethyl acetylenedicarboxylate and secondary phosphine selenide.

On the example of diethyl acetylenedicarboxylate **21a**, pyridine **1a** and bis(2-phenylethyl)phosphine selenide **2i**, it was shown that internal acetylenes of such a type were less effective in the three-component reaction with pyridines and secondary phosphine chalcogenides [49]. Therefore, at an equimolar ratio of these reagents (room temperature, 1 h) *E*-isomer of *N*-ethenyl-1,4-dihydropyridine **22** was formed in a 17% yield (Scheme 16). Under these conditions, a competitive two-component reaction of the nucleophilic addition of secondary phosphine selenide **2i** to electron-deficient acetylene **21a** mainly proceeded to give *E*-monoadduct **23**.

It is worthwhile to supplement that the phosphorylated dihydropyridines **12, 14–16** and **18** are frequent structural motifs in antihypertensive pharmaceuticals (amlodipine, nifedipine, felodipine) and prospective drug precursors in view of the similarity with known anticonvulsant [56], anticoagulant [57], antitumor [58], antitubercular [59] and cardiovascular [60] agents. The reaction found also develops the chemistry of zwitterionic adducts of pyridine to electron-deficient acetylenes, accessible reactive blocks for the synthesis of dihydropyridine derivatives [52,53,61,62,63,64,65,66] containing usually (until our investigation) 1,2-dihydropyridyl moiety.

**Scheme 17:**
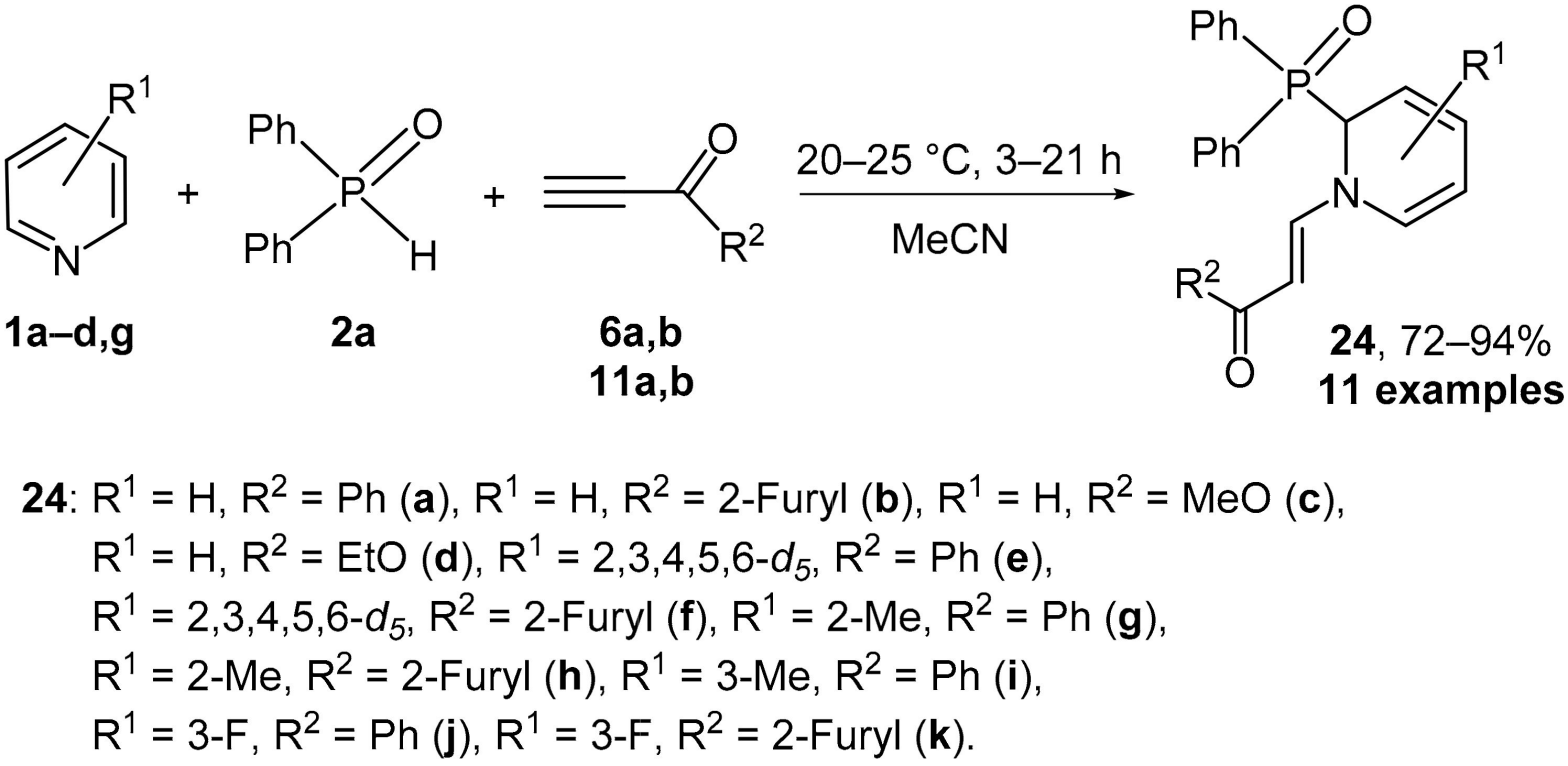
Phosphorylation/vinylation of pyridines with diphenylphosphine oxide and acylacetylenes.

In [39], it was additionally confirmed that 1-acylvinyl-2-phosphoryl-1,2-dihydropyridines **24** are kinetic products of the phosphorylation of pyridines **1** with diphenylphosphine oxide **2a** (Scheme 17).

In these reactions, the stereoselectivity is probably a result of the steric screening of position two in the *Z*-configuration of adducts **24** (Scheme 17), while this is not the case in the *E*-configuration. Finally, due to the *Z–E* equilibrium, the addition proceeds as the *E*-selective process, i.e., to form only *E*-isomers of adducts **24**.

Although the yields of dihydropyridines **24** were from good to excellent (72–94%), the reaction time differed considerably (from 3 to 21 h), indicating a significant substituents effect in the pyridine ring on the process rate. Indeed, the faster process occurred for 2- and 3-methylpyridines (4 and 3 h), whereas 3-fluoropyridine required a longer time (20–21 h). Noteworthy, the reactivity of perdeuteropyridine and non-deuterated pyridine is roughly the same that follows from close yields of the corresponding products (79–85%) and reaction times (5–5.5 h). This means that no breaking/forming of C-D bonds is involved in these steps. The more donating substituents at the P atom, as in phosphine oxide (PhCH_2_CH_2_)_2_P(O)H, preclude the phosphorylation [39] that agrees a lesser PH–acidity of these phosphine oxides [55].

Noteworthy, with terminal electron-deficient acetylenes **6, 11**, the reaction stops on kinetic *N*-vinylated/*C2*-phosphorylated intermediates. Meanwhile, with internal acetylenes **3** and **10**, the phosphorylation results in aromatization [37,38,40]. This was rationalized [37] by (i) the steric repulsion between pyridine hydrogen in position 6sixand aromatic substituent of the internal acetylene; (ii) the formation of a benzyl-like cationic intermediate; (iii) the generation of stable chalcones. Therefore, as shown above, in certain cases, the three-component reaction can be affected (delayed on the kinetic step) using terminal electron-deficient acetylenes [39,45,46,47,48].

**Scheme 18:**
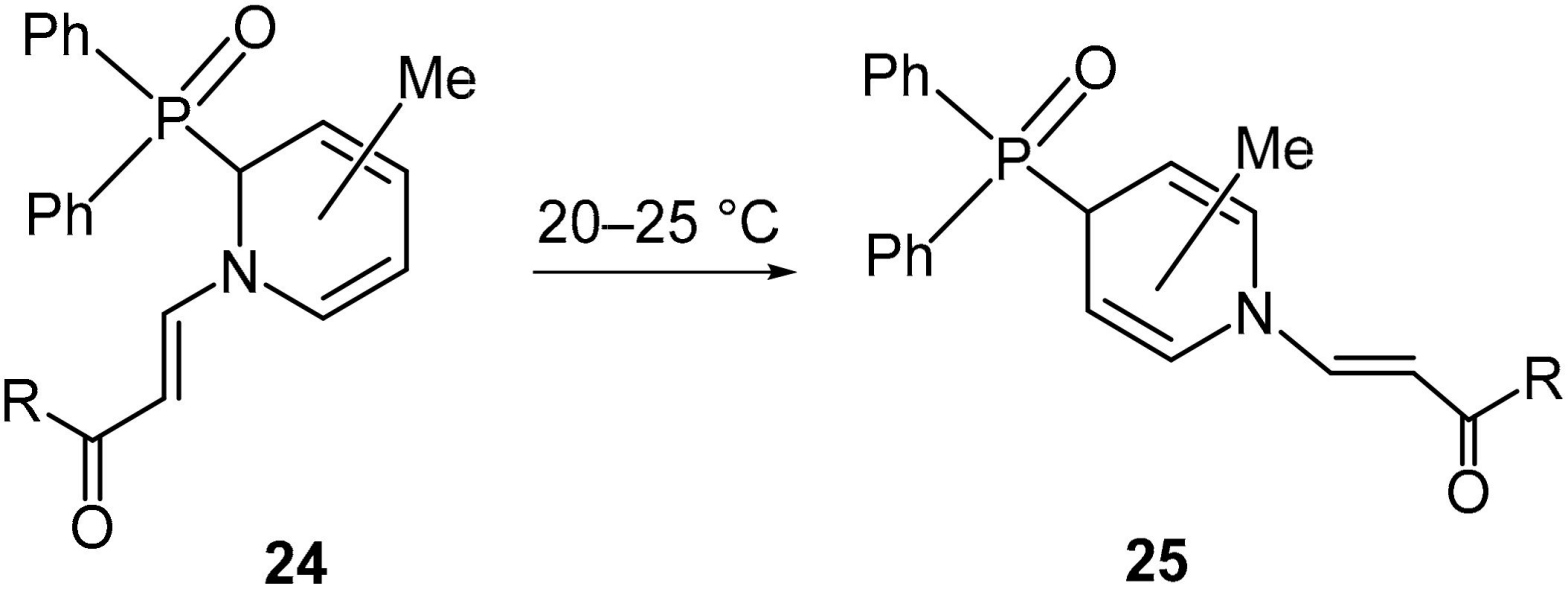
The 2→4 migration of the phosphoryl group in 1-*E*-acylvinyl-2-diphenylphosphoryldihydropyridines.

For products of methylpyridines **1b,c**, a partial 2→4 transfer of the phosphoryl groups took place [39] to give mixtures of 2- **24** and 4-regioisomers **25** of dihydropyridines in a ≈ 3–6:1 ratio (Scheme 18).

**Scheme 19:**
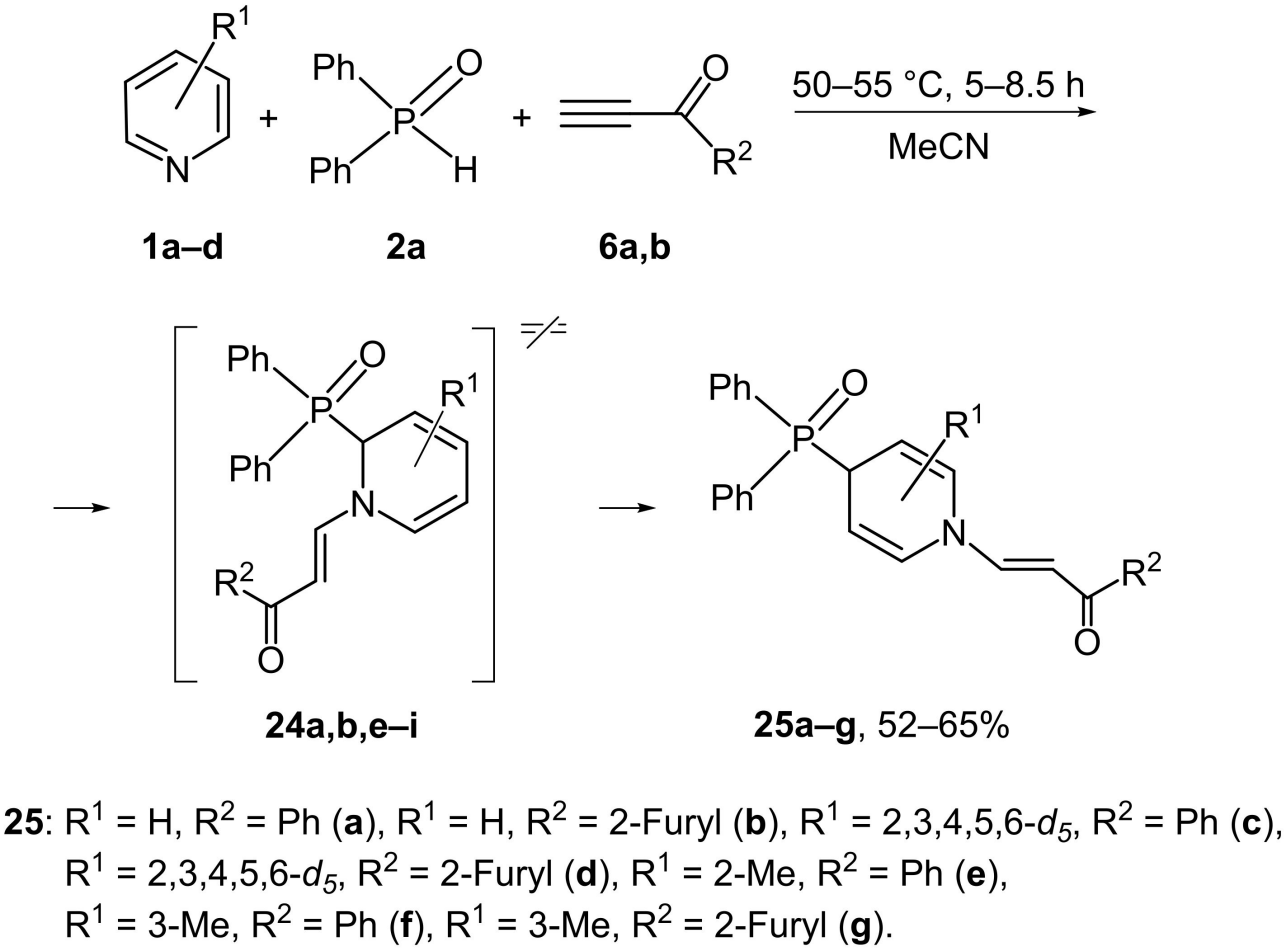
The 2→4 migration of the phosphoryl group in 1,2-dihydroadducts during the vinylation/phosphorylation of pyridines.

The rearrangement was completed when 1,2-dihydroadducts **24a,b,e–i** were heated (50–55 °C, 5–8.5 h) to produce 1,4-dihydroadducts and *E*-acylvinyl-4-phosphorylpyridines **25a–g** [39]. These products were synthesized directly from pyridines **1a–d**, phosphine oxide **2a** and acetylenes **6a,b** under the same conditions (Scheme 19) [39].

**Scheme 20:**
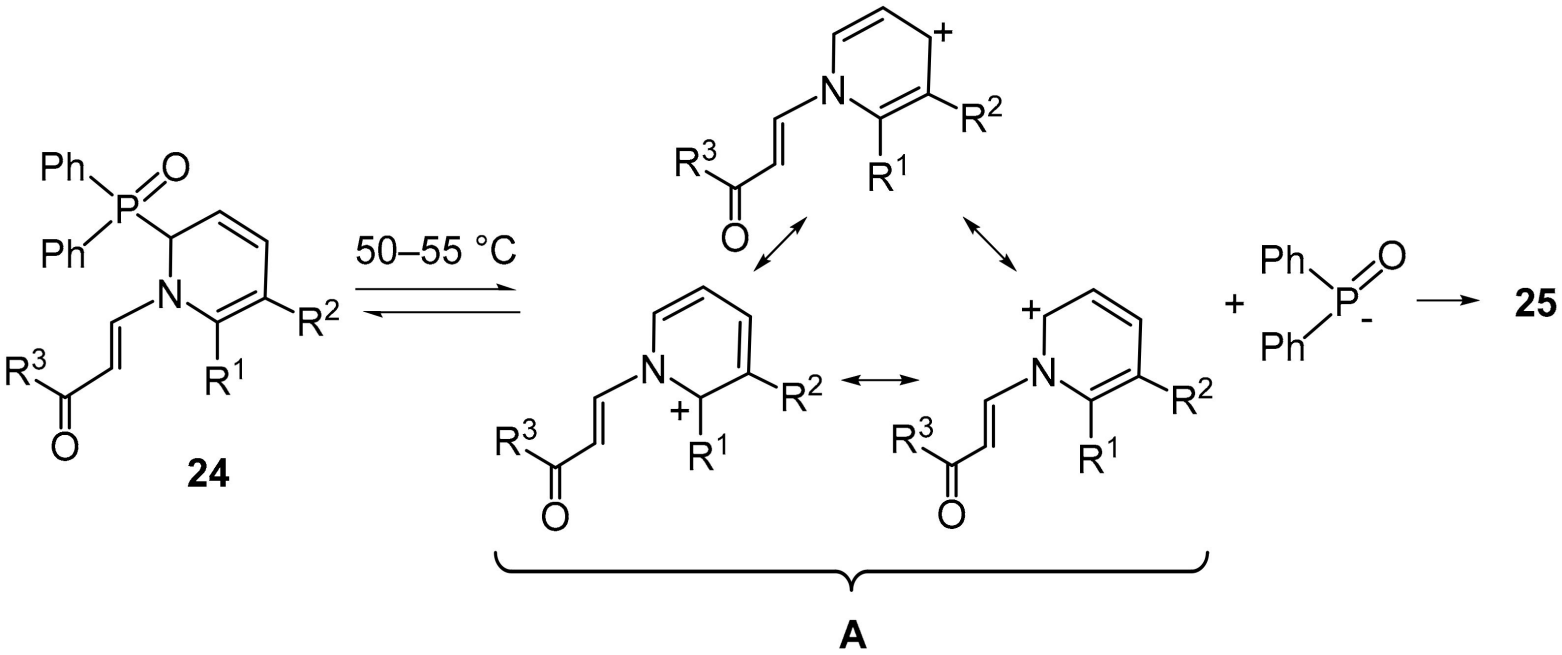
Tentative dissociation of 1,2-dihydroadducts to ion pairs.

Fluoropyridine derivatives **24j,k** turned out to be reluctantly relative to such a migration undergoing the backward aromatization to 3-fluoropyridine at a higher temperature (70–75 °C) or upon treatment with external oxidants (chloranil and DDQ). This was rationalized [39] in terms of an ion-pair interplay, including the cleavage of the C-P bond and exchange between pyridinium cation **A** and phosphine oxide anion (Scheme 20). Expectedly, the dissociation is easier the more stable the ions are (or ion-like species) formed. Consequently, the least sTable 3-fluoropyridinium cation or cation-like intermediate, due to the electron-withdrawing effect of fluorine substituent, should form in a lesser concentration, if any, and this is why the fluoro-containing 1,2-diadduct is not subjected to the rearrangement. Phosphine oxide anionic species migrate to position four to form the more thermodynamically stable 1,4-regioisomer **25**.

The higher thermodynamic stability of 1-*E*-benzoylvinyl-4-diphenylphosphoryl-1,4-dihydropyridine **25a** (by 4.0 and 3.4 kcal/mol of enthalpy in the MeCN solution and gas phase, respectively, or by 5.0 and 4.8 kcal/mol of Gibbs free energy in the MeCN solution and gas phase, respectively) compared to the corresponding 1,2-dihydropyridine **24a** was confirmed by quantum chemical calculations (B2PLYP/6-311 + G** // B3LYP/6-31 + G* + IEF PCM (B3LYP/6-31 + G*)) [39].

**Scheme 21:**
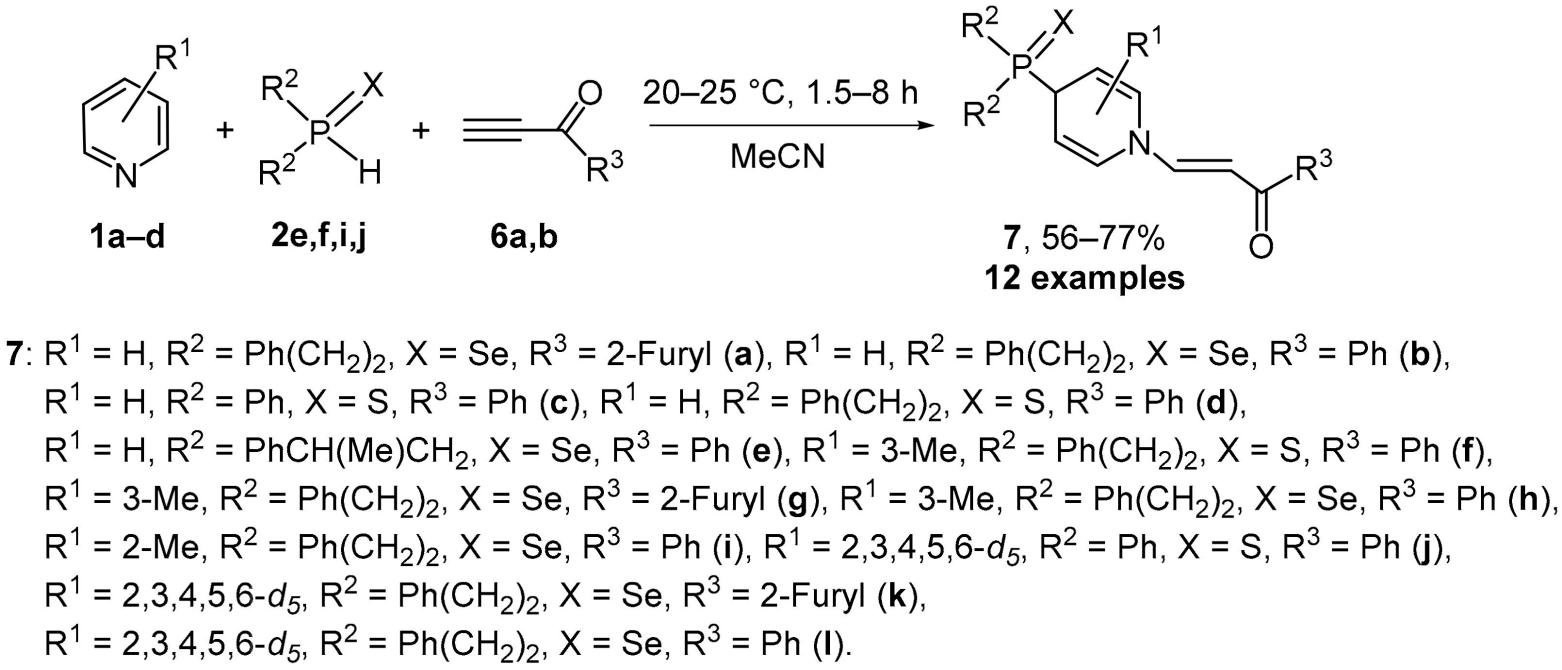
Phosphorylation/vinylation of pyridines with secondary phosphine sulfides or phosphine selenides and acylacetylenes.

Secondary phosphine sulfides **2e,f** and phosphine selenides **2i,j** reacted with pyridines **1a–d** in the presence of acylacetylenes **6a,b** (1:1:1 molar ratio) at room temperature to directly afford 1,4-dihydroadducts, 1-*E*-acylvinyl-4-thio(seleno)phosphoryldihydropyridines **7a–l** (Scheme 21) [39]. The yields for phosphine selenides **2i,j** were better than those in the case of the corresponding sulfides **2e,f**.

**Scheme 22:**
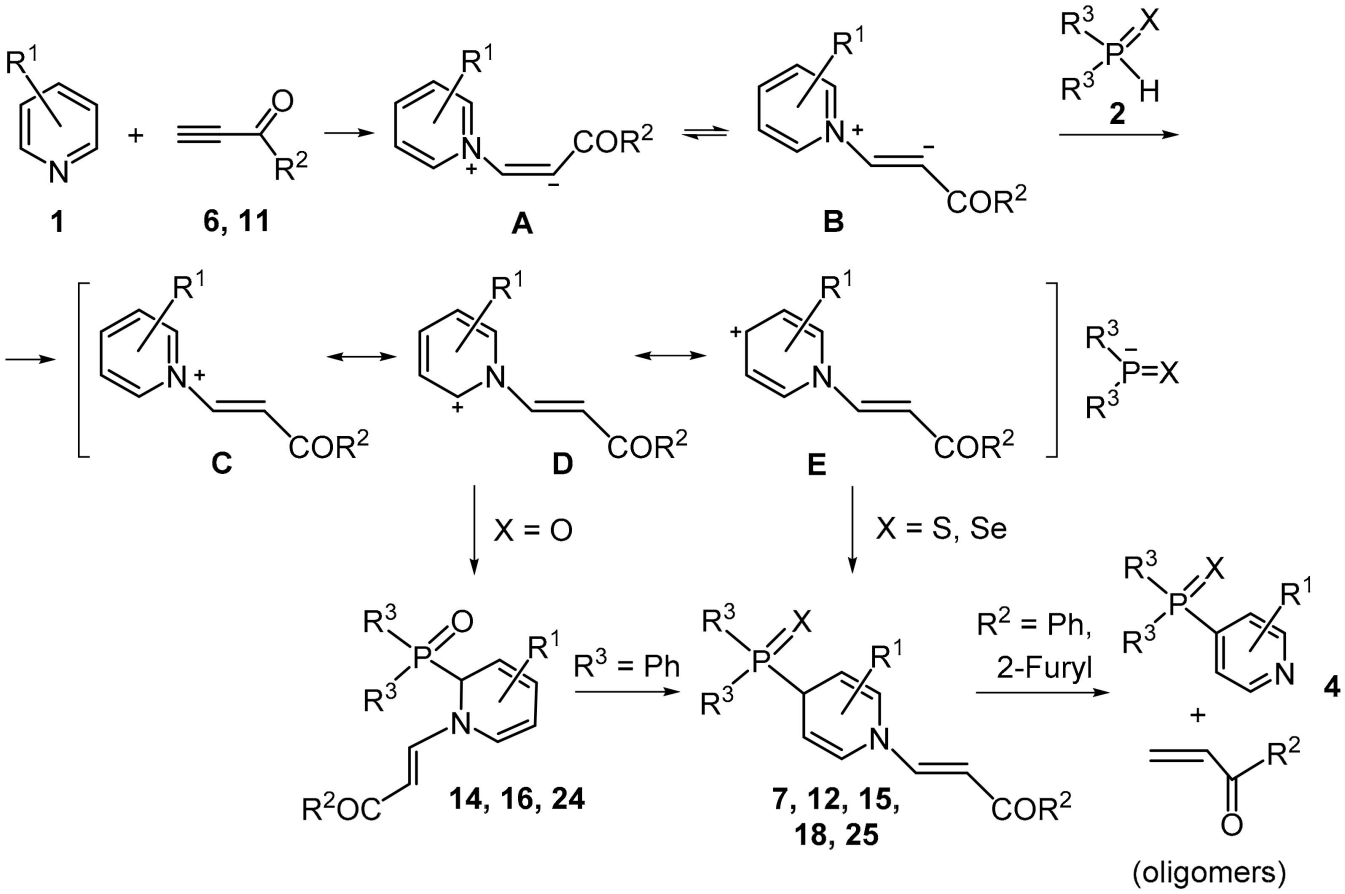
A plausible pathway of the reaction between pyridines, terminal acetylenes and secondary phosphine chalcogenides.

This version of terminal acetylene-driven **6**, **11** phosphorylation seemingly includes the generation of the intermediate dipole **A** (Scheme 22) [45]. Further, the reaction likely proceeds via the intermediate **B** as stabilized by the attractive intramolecular interaction between the carbanionic center and positively charged nitrogen atom. Proton transfer from phosphine chalcogenides to carbanionic site of zwitterion **B** leads to *N*-vinylpyridinium cation. The phosphorus-centered anion, thereby formed, attacks the position 4 of the intermediate **E** in the case of phosphine sulfides and selenides or the position 2 of the intermediate **D** when less sterically loaded phosphine oxides are used to afford the final products **7**, **12**, **15**, **18**, **25** or **14**, **16** and **24**, respectively (Scheme 22) [39,45,46,47,48,49]. Compounds **7** and **25** are capable of eliminating vinyl ketones (as oligomers) to give 4-chalcogenophosphorylpyridines **4** (Scheme 22) [39].

Noteworthy was that the known processes involving zwitterionic species resulted from the addition of pyridine to electrophilic acetylenes and the subsequent trapping of these intermediates [61] with electrophiles (activated carbon–carbon double or triple bond [62], carbonyl [63] or isocyanate groups [64], acidic CH- [65], NH- [66] or PH- [52,53] function) lead exclusively to 1,2-dihydropyridines. This implies that the attack of *P*-centered anion (Scheme 22) is directed at the alpha position of the pyridine ring.

**Scheme 23:**
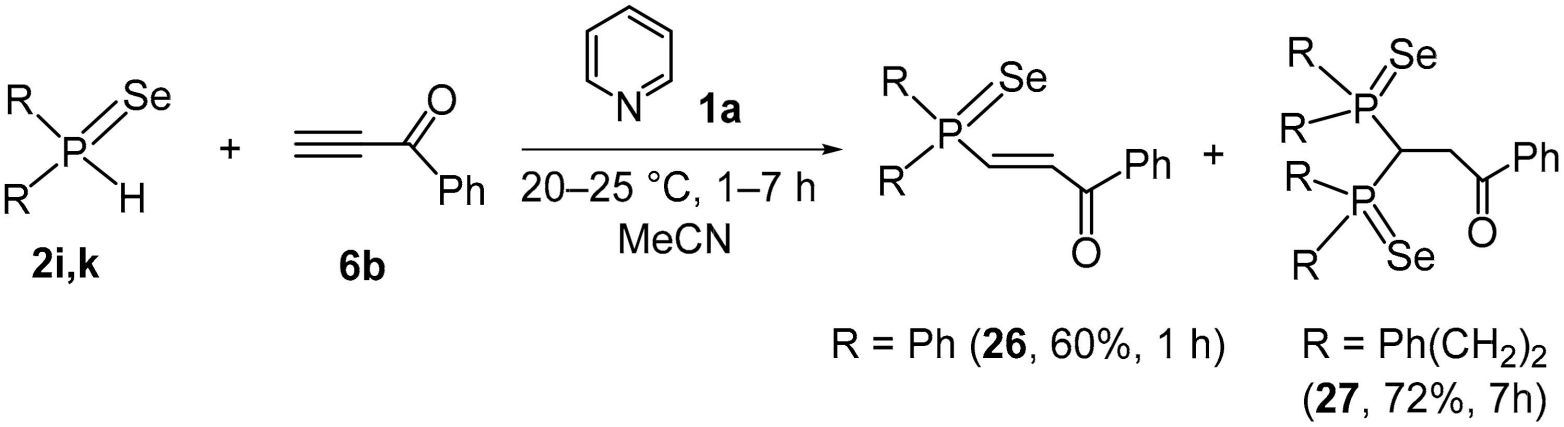
Competitive nucleophilic addition of phosphine selenides to acylacetylenes.

In the case of pyridine **1a** or 3-fluoropyridine **1g**, secondary phosphine selenides **2i,k**, and terminal acylacetylene **6b,** phosphorylation was not observed [39]. Instead, mono- **26** or diadduct **27** of phosphine selenide to acylacetylene were formed (Scheme 23).

It was suggested [39] that 3-fluoropyridine generates the initial zwitterions **A** in negligible concentration (Scheme 22), which is not enough for further phosphorylation. Overall, these results are in keeping with the above pseudo ion-pair mechanism, which implies a faster transfer of more stable chalcogenophosphoryl anions.

All the above information has evidenced two modalities of terminal acetylenes as catalysts/promoters (polarizing and deprotonating agents) in the S_N_^H^ phosphorylation of pyridines with secondary phosphine chalcogenides: (i) the repolarization of the pyridine ring rendering it a cation-like character (Scheme 22) and (ii) producing *P*-centered anions by their deprotonation of the corresponding nucleophiles (Scheme 22). This gave the key intermediates, 2- and 4-phosphorylated acylvinylpyridines **24**, **25** and **7** (Scheme 17, Scheme 19 and Scheme 21). The third modality of these electron-deficient acetylenes, i.e., to behave as inner oxidants, readily occurs at a high temperature.

**Scheme 24:**
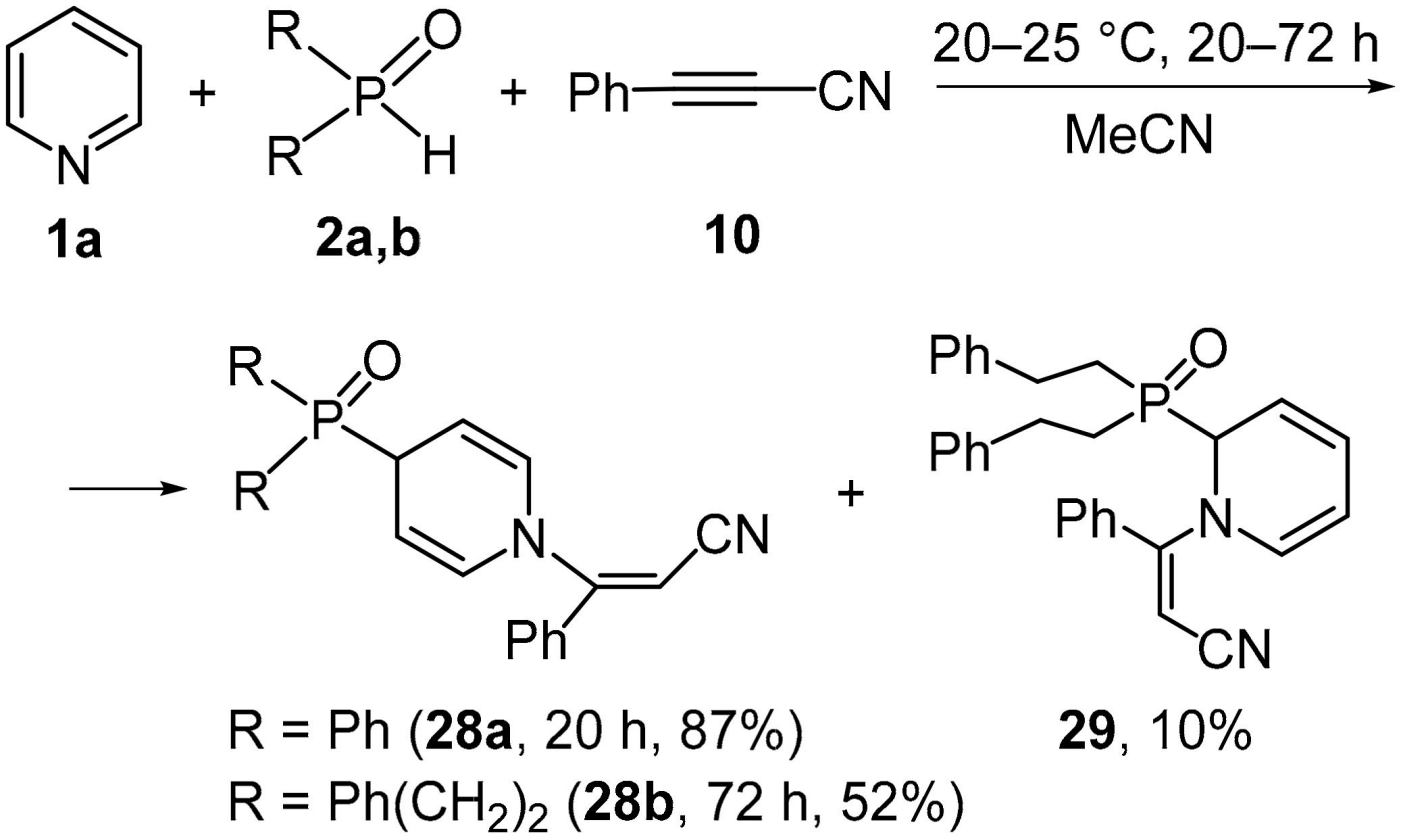
Three-component reaction between 3-phenyl-2-propynenitrile, secondary phosphine oxides and pyridine.

Likewise, another electron-deficient acetylene, 3-phenyl-2-propynenitrile **10**, acted as an adjuvant in phosphorylation of pyridines (ambient temperature) with phosphine oxides **2a,b** to regio- and stereoselectively afford (*Z*)-*N*-(2-cyano-1-phenyl)ethenyl-4-phosphoryl-1,4-dihydropyridines **28a,b**, key intermediates of the delayed S_N_^H^ reaction, in a 87% and 52% yield [40]. Only with phosphine oxide **2b** was the formation of 1,2-dihydropyridine **29** in a 10% yield observed (Scheme 24).

Interestingly, 1,2-dihydropyridine **29** happened to be stable; the expected 2→4-migration [39] of the phosphoryl substituent did not take place even at a higher temperature (70–75 °C).

As it was shown above (see Section 2.1, Scheme 8), the anticipated S_N_^H^ phosphorylation of pyridines was realized when the reactants were heated up to 85 °C; both 1,2- and 1,4-dihydropyridine intermediates gave 4-phosphorylpyridines **4a,b** in 30–35% yields [40] with the elimination of 3-phenyl-2-propenenitrile oligomers (Scheme 8).

This S_N_^H^ phosphorylation was assumed to follow the mechanism shown in Scheme 22, i.e., via the reversible generation of zwitterions **A–B**, intermediate cations **C–E** and phosphorus-centered anions [45]. The latter is attached to either position four of the intermediate **E** pyridinium ring in the case of phosphine oxide **2a** or position two of the intermediate **D** with less spatially encumbered phosphine oxide **2b** to afford the final products **28a,b** and **29**. Finally, dihydropyridine formed oxidatively (relative to dihydropyridine moiety) releases (upon heating) phenylpropenenitrile as oligomers to deliver S_N_^H^ substitution products **4**.

### 2.3. Phosphorylation of Pyridines with H-Phosphonates in the Presence of Electron-Deficient Acetylenes

**Scheme 25:**
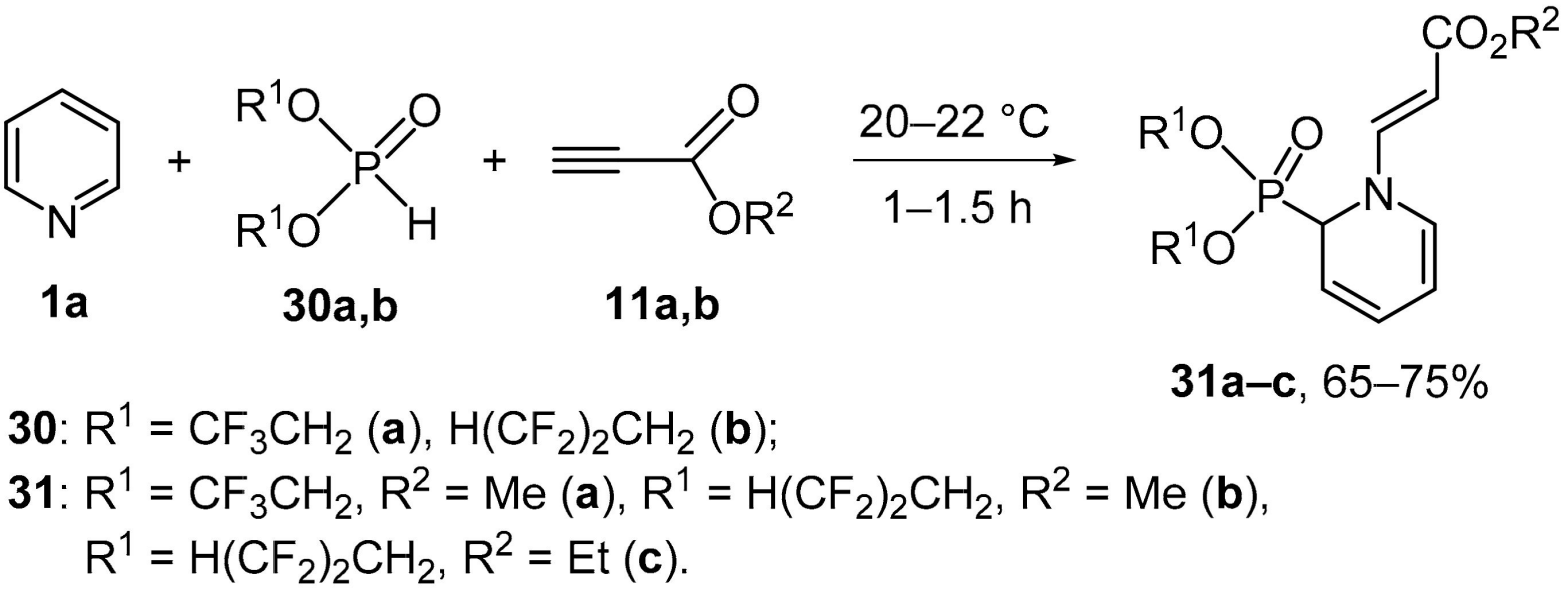
Catalyst- and solvent-free *N*-vinylation/*C*(2)-phosphorylation of pyridine with alkyl propiolates and bis(fluoroalkyl) *H-*phosphonates.

The S_N_^H^ phosphorylation of pyridine **1a** with bis(fluoroalkyl) *H-*phosphonates **30** in the presence of alkyl propiolates **11** retained on the formation of intermediate 1,2- and 1,4-dihydropyridines. With bis(2,2,2-trifluoroethyl) or bis(2,2,3,3-tetrafluoropropyl) *H-*phosphonates **30a,b**, the phosphorylation (ambient temperature, no solvent) regio- and stereoselectively led to (*E*)-*N*-alkoxycarbonylethenyl-1,2-dihydropyridines **31a–c** in a 65–75% yield (Scheme 25) [67].

**Scheme 26:**
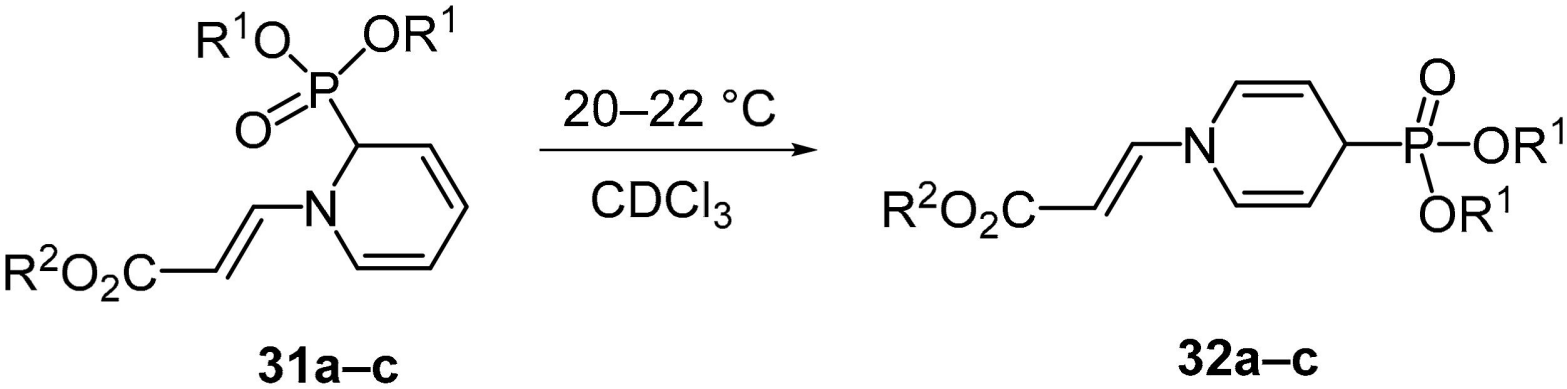
Rearrangement of functional 1,2-dihydropyridines to the 1,4-isomers.

In the solid state, these 1,2-dihydropyridines **31a–c** were stable at ambient temperature. However, in CDCl_3_, they rearranged to the corresponding 1,4-dihydropyridines **32a–c** (Scheme 26) [67].

**Scheme 27:**
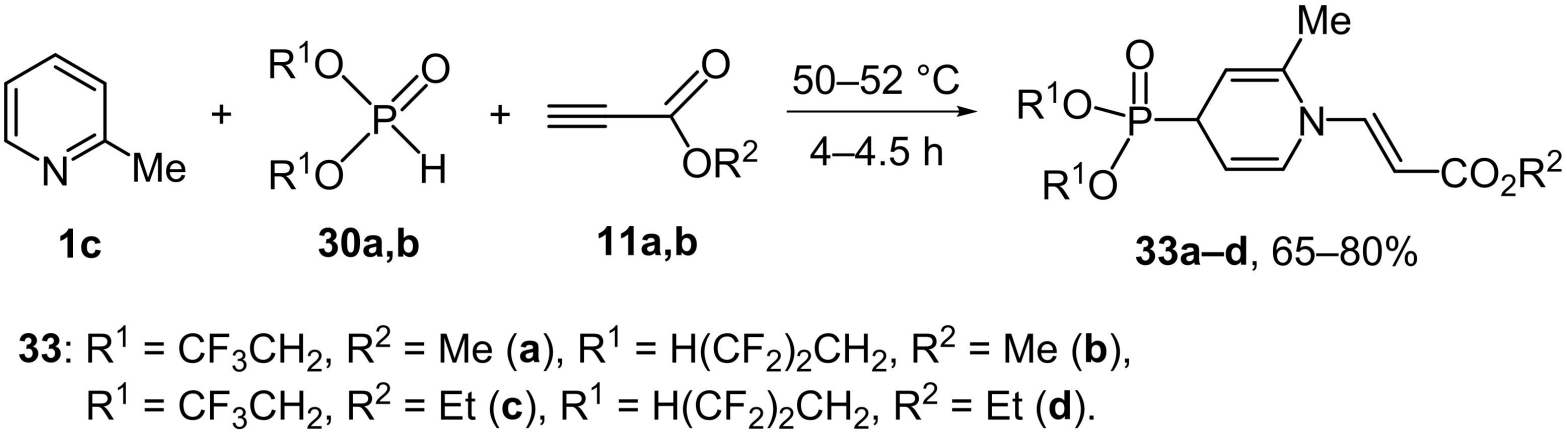
Catalyst- and solvent-free *N*-vinylation/*C*(4) phosphorylation of 2-methylpyridine with alkyl propiolates and bis(fluoroalkyl) *H-*phosphonates.

Noteworthy, the solvent-free phosphorylation of 2-methylpyridine **1c** with the same fluorinated *H*-phosphonates **30a,b** and alkyl propiolates **11a,b** proceeded upon heating at 50–52 °C to furnish 1,4-dihydropyridines **33a–d** in a 65–80% yield (Scheme 27) [67].

This reaction at room temperature lasted longer (20 h vs. 4–4.5 h) to form phosphonylated 1,2-dihydropyridines in a small concentration. This confirms that this step is a kinetic one [67].

**Scheme 28:**
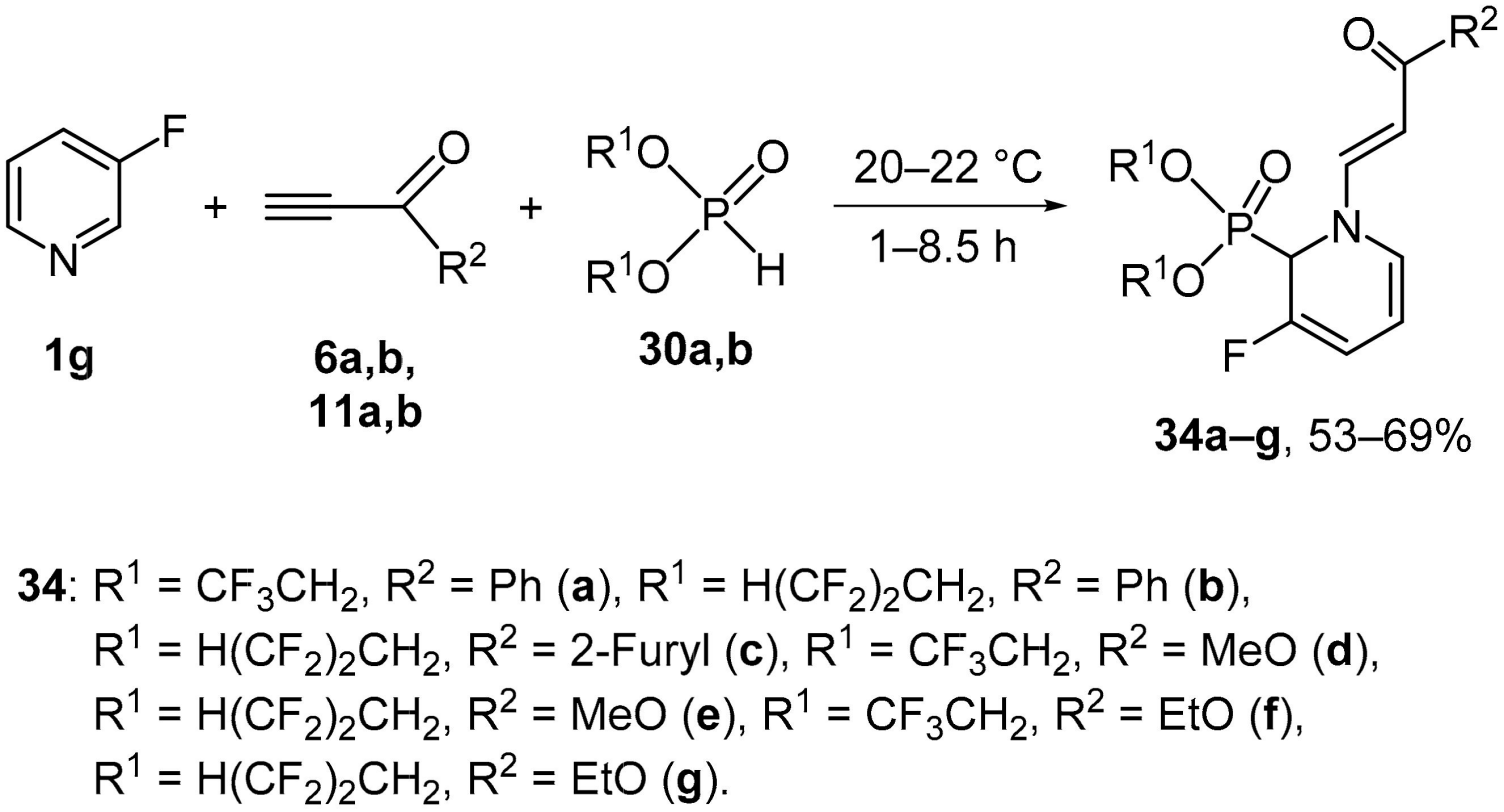
Three-component reaction between 3-fluoropyridine, electron-deficient alkynes and bis(fluoroalkyl) *H*-phosphonates.

Under similar conditions (20–22 °C, 1–8.5 h and solvent-free), phosphonylated 1,2-dihydropyridines **34a–g** were obtained from 3-fluoropyridine **1g**, electron-deficient alkynes (alkyl propiolates **11a,b** or acylacetylenes **6a,b**) and bis(fluoroalkyl) *H-*phosphonates **30a,b** (Scheme 28) [68].

**Scheme 29:**
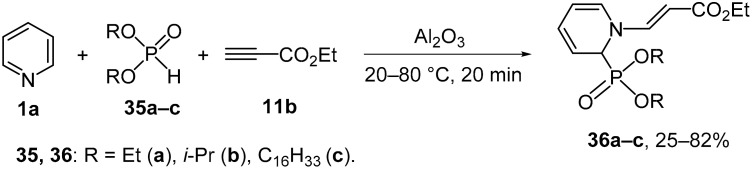
Catalyst reaction between pyridines, alkyl propiolates and dialkyl *H*-phosphonates.

Earlier [52], the reaction of pyridine **1a**, ethyl propiolate **11b** and dialkyl *H*-phosphonates **35a–c** in the presence of Al_2_O_3_ as a catalyst was shown to afford 1,2-dihydropyridine phosphonates **36a–c** (Scheme 29).

**Scheme 30:**
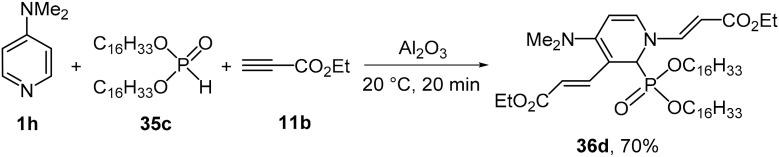
Catalyst reaction between DMAP, ethyl propiolate and dialkyl *H*-phosphonate.

The outcome of the reaction depends on the pyridine ring substitution. Therefore, no reaction occurred with 2,6-1utidine, whereas, in the case of 4-dimethylamino-pyridine **1h** (DMAP), 1,2-dihydropyridine phosphonate bis-adduct **36d**, resulting from the addition of two ethyl propiolate moieties to the pyridinium ring, was obtained (Scheme 30) [52]. The electrophilic character of the unsaturated substrate was also important, since authors have never observed any of the expected reactions with acrylonitrile or ethyl acrylate. The dry medium process enhanced the addition rate and improved the yield with respect to the homogeneous medium.

Thus, the results presented in Section 2.1, Section 2.2 and Section 2.3 indicate that the reactions of pyridines with PH–nucleophiles (phosphine chalcogenides, *H*-phosphonates) triggered and driven by electron-deficient acetylenes open convenient access to in-demand phosphorylated pyridines, the target S_N_^H^ products and (or) deeply functionalized dihydropyridines, important intermediates for S_N_^H^ phosphorylation.

## 3. S_N_^H^ Phosphorylation of Quinolines and Isoquinolines

**Scheme 31:**
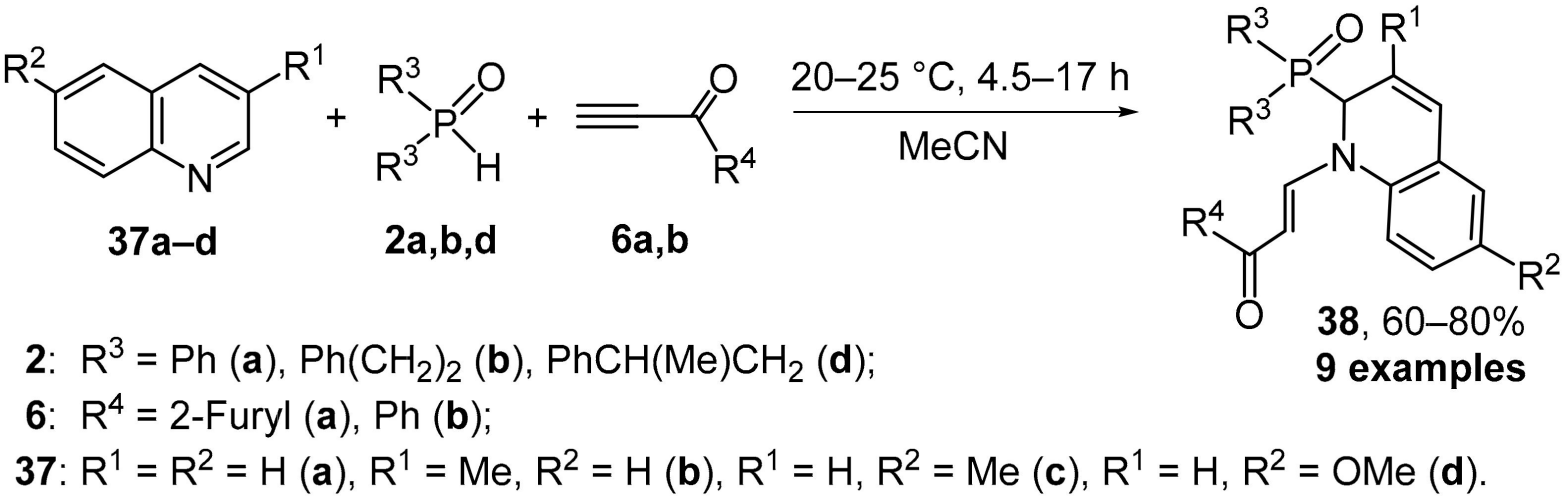
Tandem regio- and stereoselective addition of acylacetylenes and secondary phosphine oxides to quinolines.

Quinolines **37a–d** reacted with secondary phosphine oxides **2a,b,d** and terminal acylacetylenes **6a**,**b** (room temperature) [69] to yield *N*-acylvinyl-2-phosphoryldihydroquinolines **38** (Scheme 31), i.e., here, the first step of S_N_^H^ phosphorylation (the reductive insertion of phosphine oxides **2a,b,d** into the heterocyclic core) occurred.

Under these conditions, oddly, only 1,2-adducts of acylacetylenes and phosphine oxides to quinolines were regioselectively formed with a complete regioselectivity of the enone moiety. All of these, together with much milder conditions, differ this reaction from that with pyridines [39]. Thus, the reaction stopped at the formation of the dihydro intermediates, i.e., a delayed S_N_^H^ reaction took place here.

**Scheme 32:**
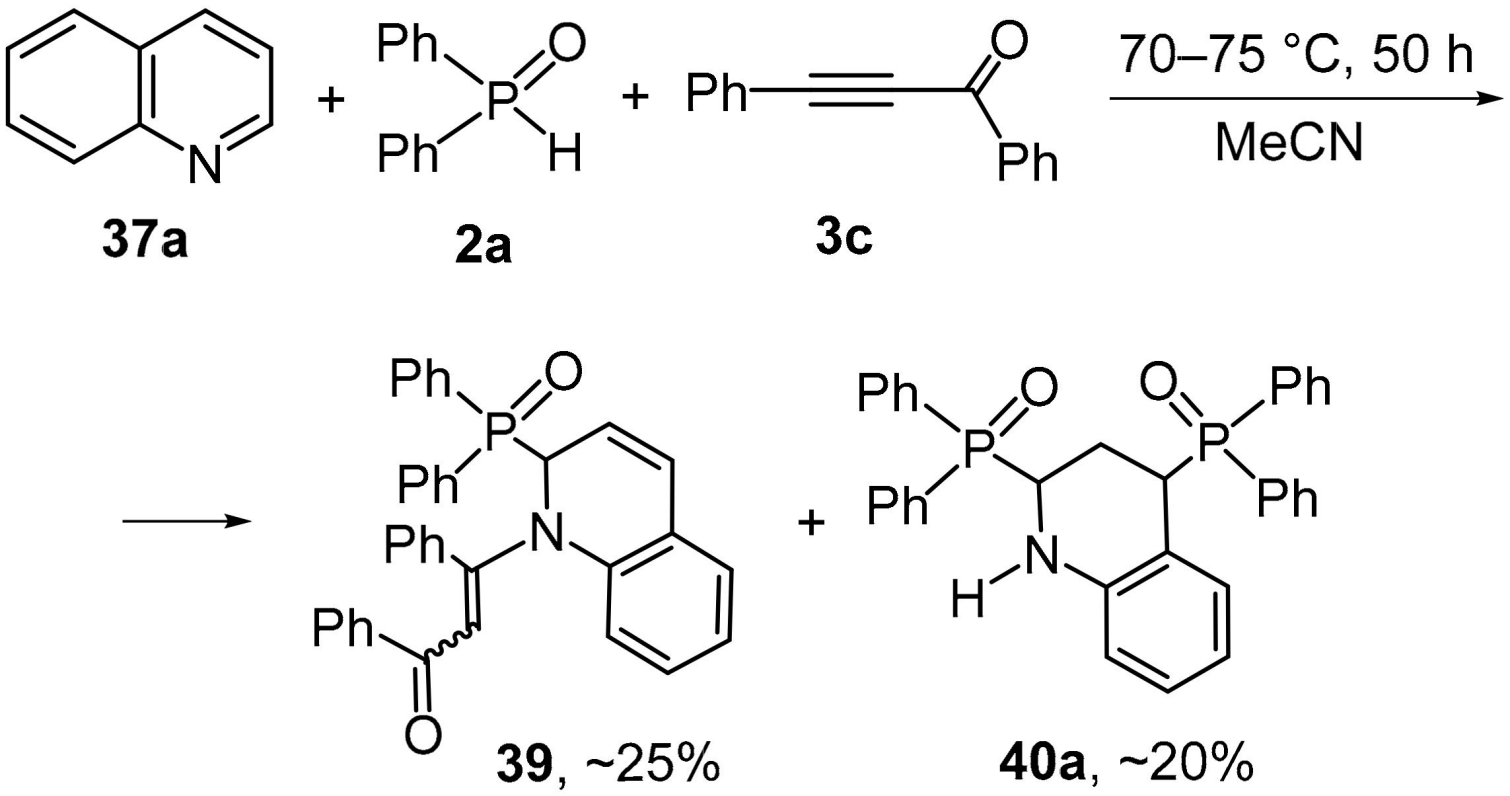
Reaction of benzoylphenylacetylene and diphenylphosphine oxide with quinoline.

The longest duration of the process (17 h) was observed for the bulkiest nucleophile (phosphine oxide **2d**). The decisive role of the steric effect in such a reaction was noted for an internal acylacetylene, i.e., benzoylphenylacetylene **3c**; the reaction occurred at 70–75 °C for 50 h to give the expected *N*-benzoylvinyl-2-diphenylphosphoryldihydroquinoline **39** and, unexpectedly, 2,4-bis(diphenylphosphoryl)tetrahydroquinoline **40a** (Scheme 32), the latter being obviously formed without acylacetylene **3c** [69].

**Scheme 33:**
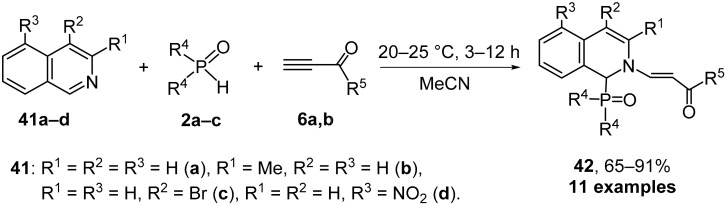
Tandem regio- and stereoselective addition of acylacetylenes and secondary phosphine oxides to isoquinolines.

The reaction of isoquinolines **41a–d** with secondary phosphine oxides **2a–c** and terminal acylacetylenes **6a,b** (room temperature, MeCN) was faster (3–12 h) and provided better yields (**42**, up to 91%) as compared to the quinolines (Scheme 33) [69].

The slowest phosphorylations (10, 12 h) were for 4-bromo- and 5-nitroisoquinolines **41c,d**, particularly when bulkier bis(2-phenylethyl)phosphine oxide **2b** was employed, confirming the considerable contribution of the nitrogen atom basicity and steric screening of the phosphorus-centered anion to the reaction outcome. Evidently, the process is accelerated when isoquinoline basicity increases and steric constrains from the phosphine oxide side reduce.

**Scheme 34:**
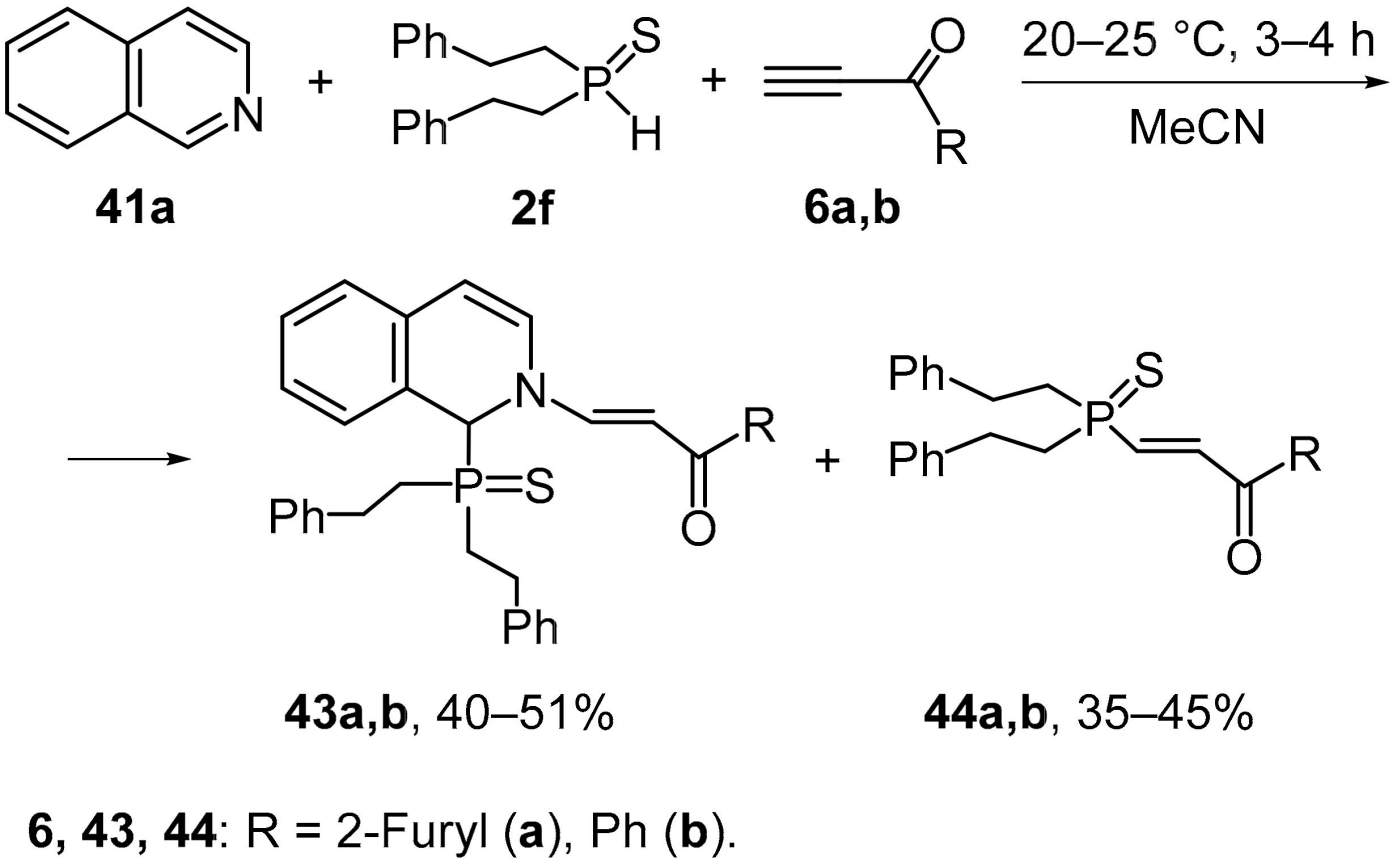
Reaction of isoquinoline, bis(2-phenylethyl)phosphine sulfide and acylacetylenes.

Noteworthy, the reaction with bis(2-phenylethyl)phosphine sulfide **2f** gave, together with the anticipated *N*-acylvinyl-1-phosphorylated dihydroisoquinolines **43a,b**, the adducts **44a,b** of this phosphine chalcogenide to acylacetylenes (Scheme 34) [69].

**Scheme 35:**
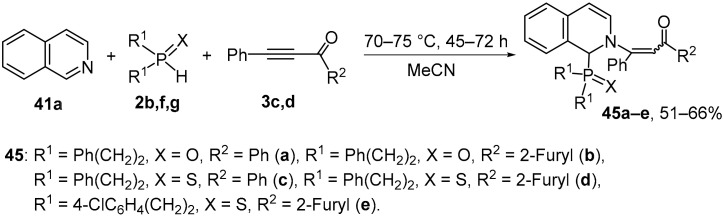
Tandem addition of acylphenylacetylenes and secondary phosphine chalcogenides to isoquinoline.

The key steric influence on this tandem vinylation/phosphorylation of isoquinoline was particularly displayed in the reaction with internal acetylenes **3c**,**d**. In this case, even at a higher temperature (70–75 °C), the process was about ten times slower and the stereoselectivity was lost; the reaction mixture contained 70–75% of the **45** *Z*-isomer, which was proved to be a kinetic product (Scheme 35).

**Scheme 36:**
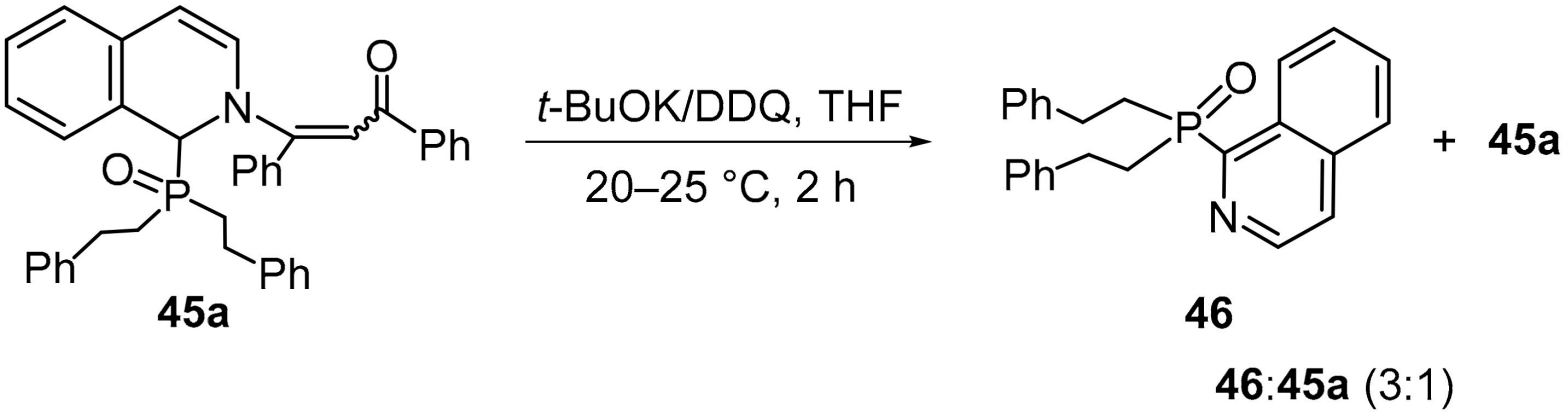
Aromatization of dihydroisoquinoline by *t*-BuOK/DDQ system.

Common oxidants (chloranil or DDQ) induced [69] the retroaromatization to isoquinolines with the elimination of phosphine oxide **2b**, which was fixed by quinones. The same transformation was observed for dihydroisoquinoline **45a** under the action of *t*-BuOK in THF. The combination of *t*-BuOK with DDQ allowed reaching a positive result in the completion of the S_N_^H^ reaction; the expected aromatic bis(2-phenylethyl)phosphorylisoquinoline **46** was detected among the reaction products along with the initial compound **45a** (Scheme 36).

**Scheme 37:**
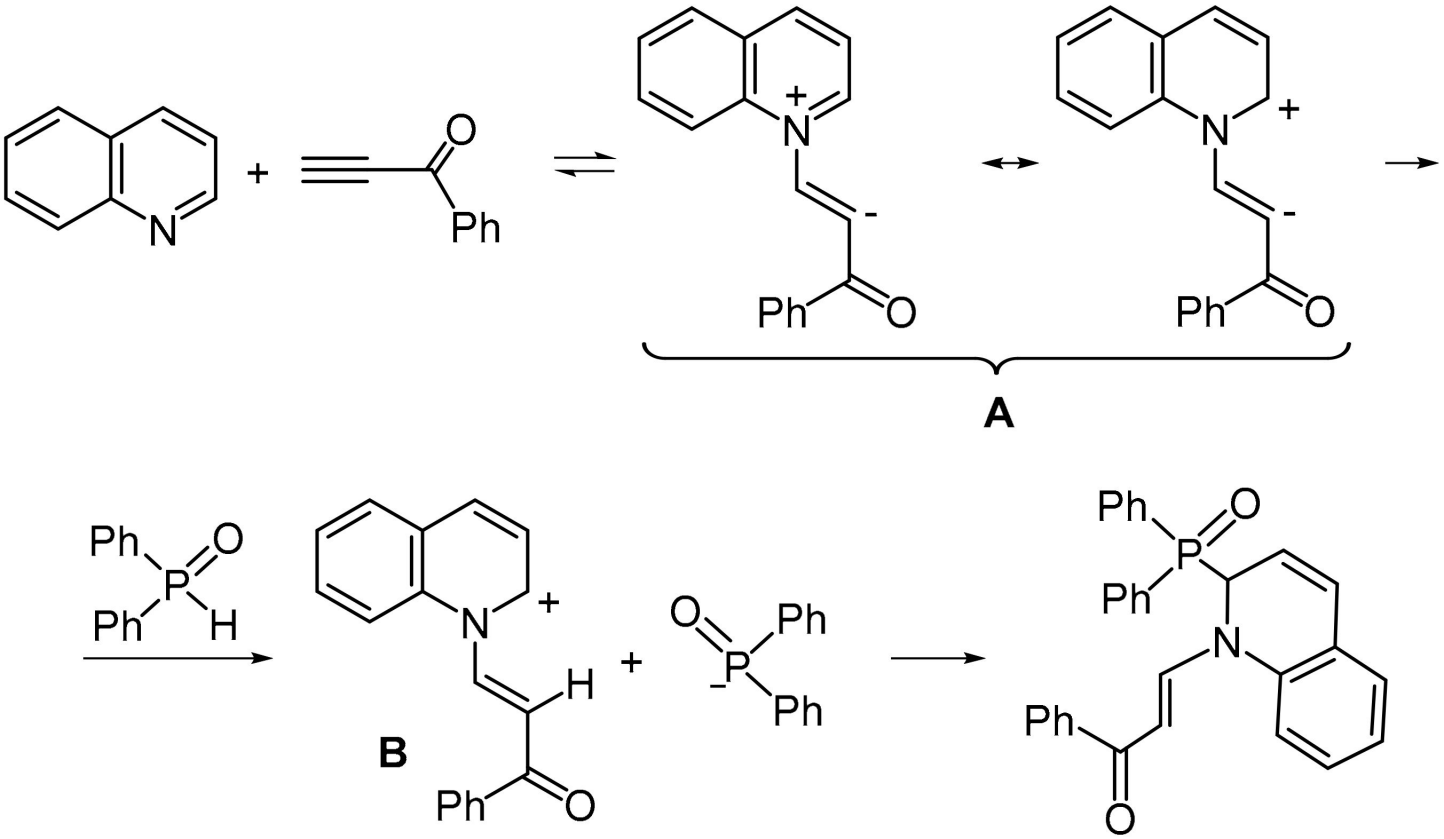
Plausible mechanism of tandem addition of acylacetylenes and secondary phosphine chalcogenides to (iso)quinolines.

It is believed [69] that the above phosphorylation is triggered by acylacetylenes, which form with quinolines (isoquinolines) 1,3(4)-dipolar intermediate **A**. A further hydrogen transfer from phosphine chalcogenides to the carbanionic site of intermediate **A** occurs and the carbocationic intermediate **B**, thus produced, accepts the phosphorus-centered anion onto position two of the quinoline moiety to give the final products (Scheme 37).

This mechanism is consistent with the experimental fact that the reaction with isoquinolines is more facile than that with quinolines; a higher basicity of isoquinoline compared to quinolines (p*K*_a_ values 5.46 and 4.93, respectively) ensures a greater concentration of triggering intermediate **A** (Scheme 37). Thus, electron-withdrawing substituents in the isoquinoline ring slow down the reaction (Scheme 33). Additionally, a bulkier phosphine oxide **2b** and internal acylacetylenes **3c,d** significantly hamper the reaction due to steric hindrance for the generation of intermediates **A** and **B**. Consequently, the formation of *E*-isomers (with terminal acylacetylenes) and kinetic *Z*-isomers (in the case of internal acetylenes) is an expected result of steric prerequisites. The reaction regioselectivity is understood in terms of an anticipated stronger positive charge at the α position relative to the nitrogen atom, provided the process is a charge-controlled one [69].

The quantum chemical insight [69] (HF/6-311G**//B3LYP/6-311G**) reveals that positions two both in pyridine- and quinoline zwitterions are positively charged, while positions four are almost neutral. Meanwhile, the LUMO localization in position four of pyridine is higher than that in quinoline, the α positions have a much lower LUMO localization. It follows that the phosphorylation of pyridines activated by acylacetylenes is orbital controlled, while a similar reaction in quinoline or isoquinoline series depends on charge distribution. This theoretical assessment helps to understand the different behavior of pyridines and quinolines (isoquinolines) in acetylene-triggered/driven S_N_^H^ phosphorylation; the complete aromatic substitution for the former and the delayed process on the dihydrointermediate step for the latter.

**Scheme 38:**
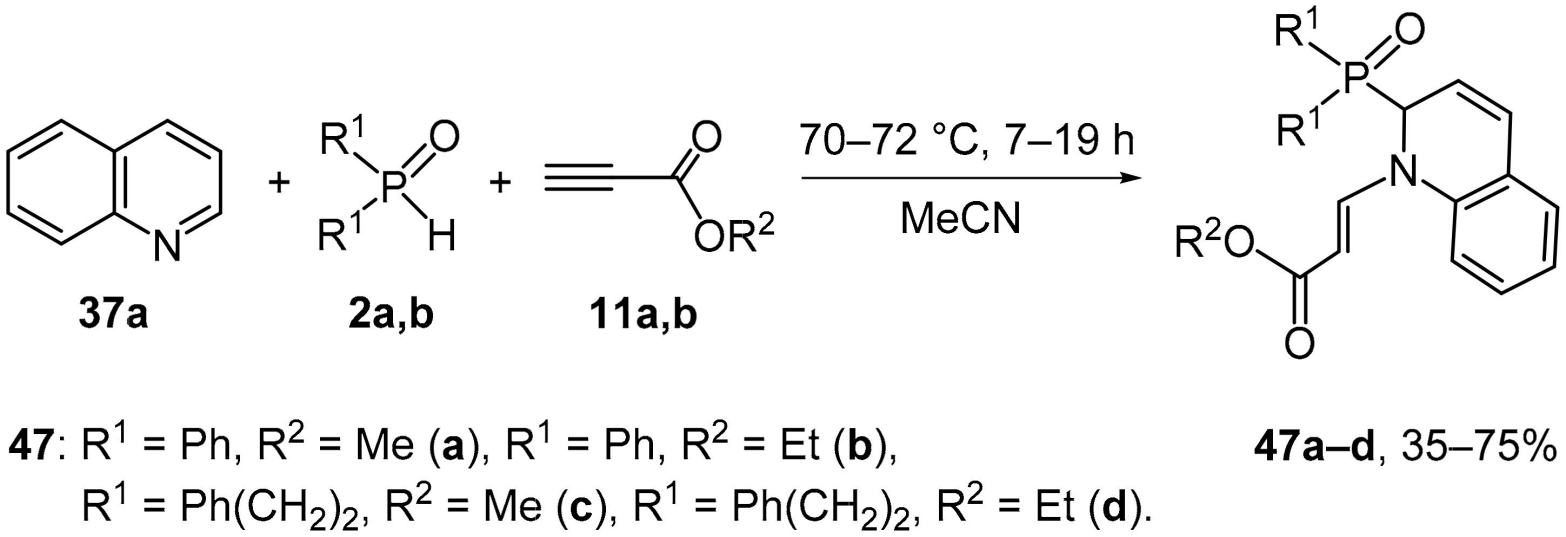
*N*-vinylation/*C*-phosphorylation of quinoline with alkyl propiolates and secondary phosphine oxides.

Alkyl propiolates **11a,b** triggered and further driven S_N_^H^ phosphorylation (70–72 °C, 7–19 h) of quinoline **37a** with secondary phosphine oxides **2**, which, such as the reaction with acylacetylenes, stopped on the formation of the intermediate dihydroquinolines **47a–d** with alkyl propiolate substituents at the N atom (Scheme 38) [70].

**Scheme 39:**
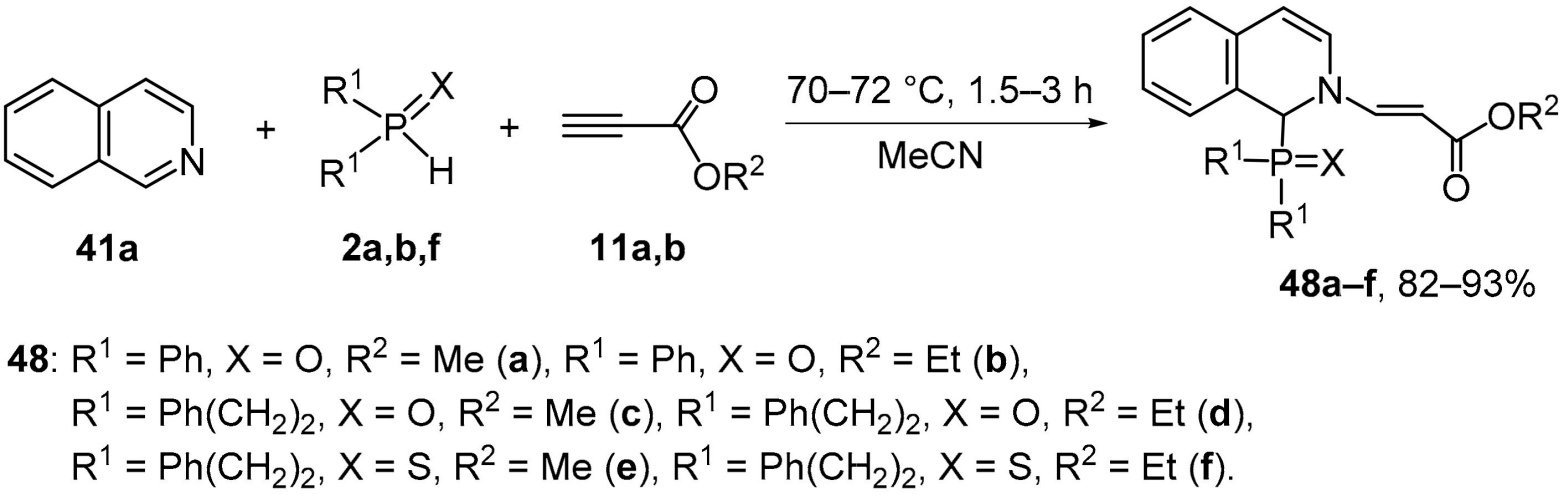
*N*-vinylation/*C*-phosphorylation of isoquinoline with alkyl propiolates and secondary phosphine chalcogenides.

With isoquinoline **41a,** the reaction was more facile, taking 1.5–3 h, and more efficient to afford 1,2-dihydroisoquinolines **48a–f** in up to a 93% yield (Scheme 39) [70]. In both cases (Scheme 38 and Scheme 39), the reaction was regio- and stereoselective providing 1,2-dihydroisomers of the *E*-configuration relative to the double bond.

**Scheme 40:**
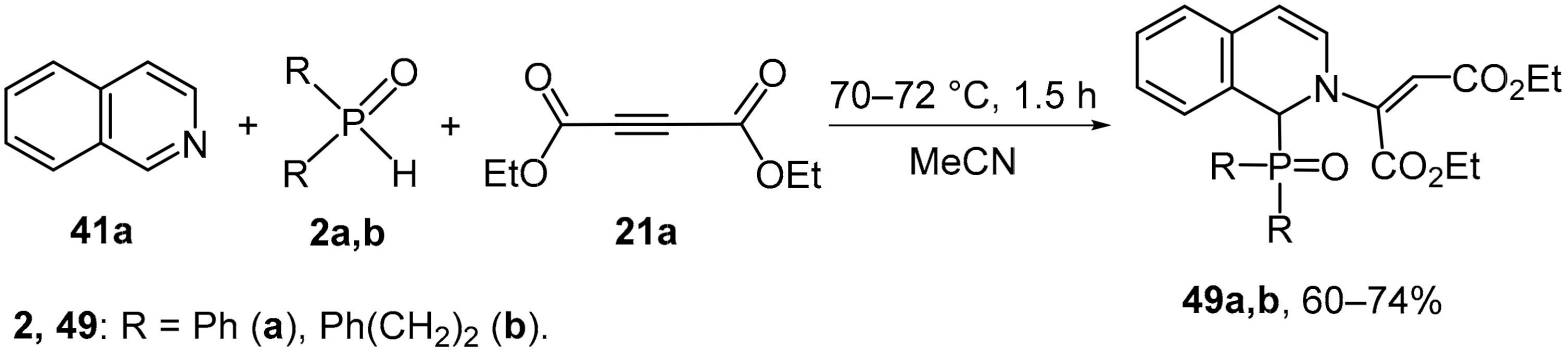
*N*-vinylation/*C*-phosphorylation of isoquinoline with acetylenedicarboxylate and secondary phosphine oxides.

The same phosphorylation of isoquinoline **41a** with secondary phosphine oxides **2a,b** in the presence of diethyl acetylenedicarboxylate **21a** as an adjuvant regio- and stereoselectively yielded (*E*)-*N*-ethenyl-1,2-dihydroisoquinolines **49a**,**b** (Scheme 40) [70].

**Scheme 41:**
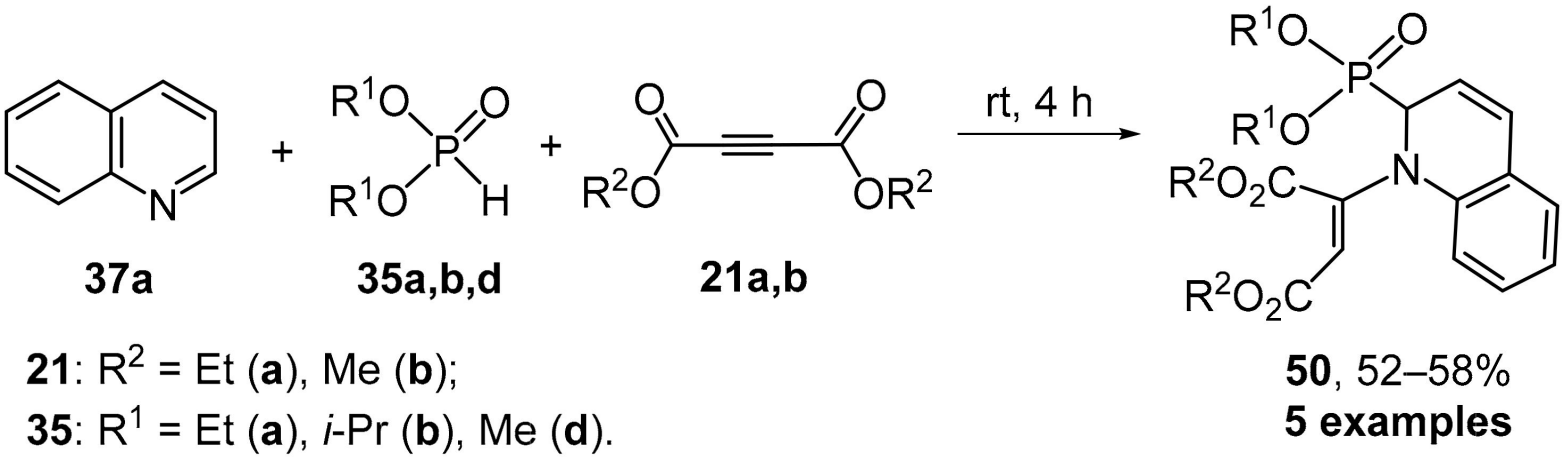
*N*-vinylation/*C*-phosphorylation of quinoline with acetylenedicarboxylates and dialkyl *H*-phosphonates.

In the work [53], a three-component reaction between quinoline **37a**, dialkyl *H*-phosphonates **35a,b,d** and acetylenedicarboxylates **21a,b**, providing phosphonylated 1,2-dihydroquinolines **50**, was reported (Scheme 41). The reaction was stereoselective; the final products were mainly of the *E*-configuration.

**Scheme 42:**
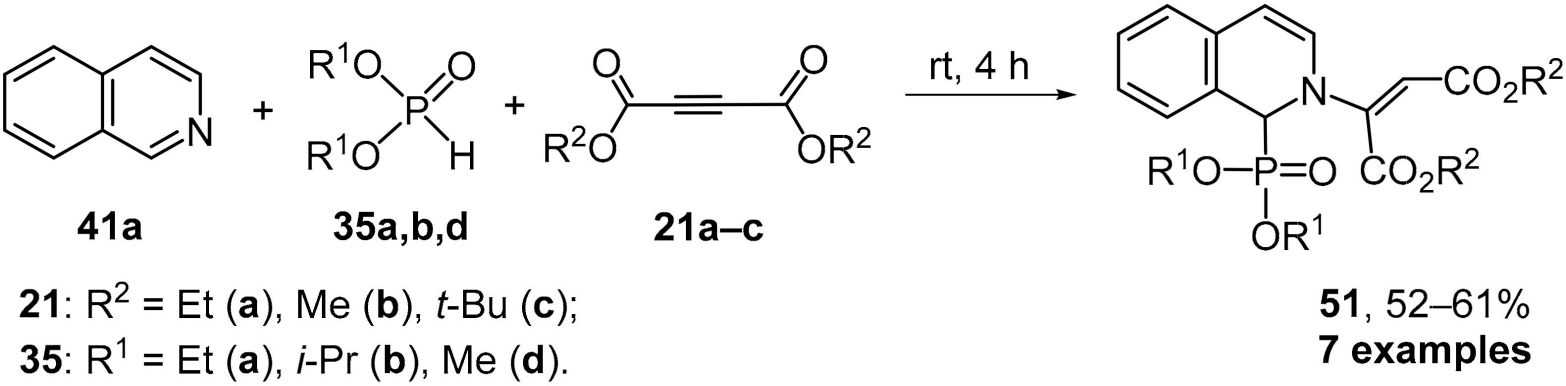
*N*-vinylation/*C*-phosphorylation of isoquinoline with acetylenedicarboxylates and dialkyl *H*-phosphonates.

The authors also show [53] that isoquinoline **41a** reacted with *H*-phosphonates **35a,b,d**, and acetylenedicarboxylates **21a–c** under solvent-free conditions at room temperature to give 1,2-dihydroisoquinolin-1-ylphosphonate derivatives **51** in good yields (Scheme 42).

**Scheme 43:**
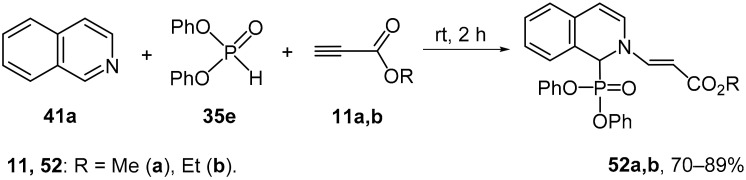
*N*-vinylation/*C*-phosphorylation of isoquinoline with alkyl propiolates and diphenyl *H*-phosphonate.

**Scheme 44:**
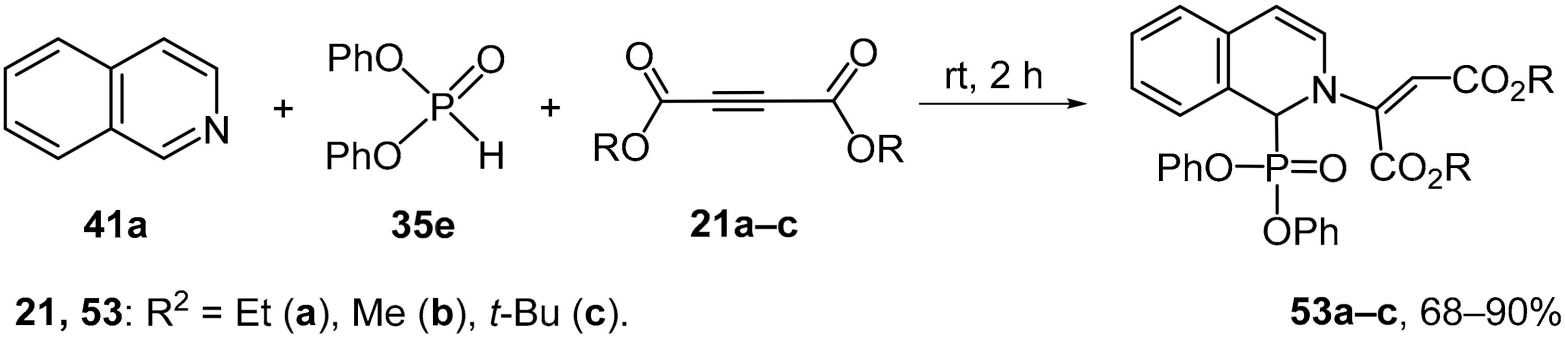
*N*-vinylation/*C*-phosphorylation of isoquinoline with acetylenedicarboxylates and diphenyl *H*-phosphonate.

The reaction of isoquinoline **41a** and electrophilic acetylenes **11a,b** and **21a–c** in the presence of diphenyl *H*-phosphonate **35e** proceeded smoothly (rt, 2h) to deliver the corresponding 1,2-dihydroisoquinoline phosphonates **52a,b** or **53a–c** in a 68–90% yield (Scheme 43 and Scheme 44) [71].

**Scheme 45:**
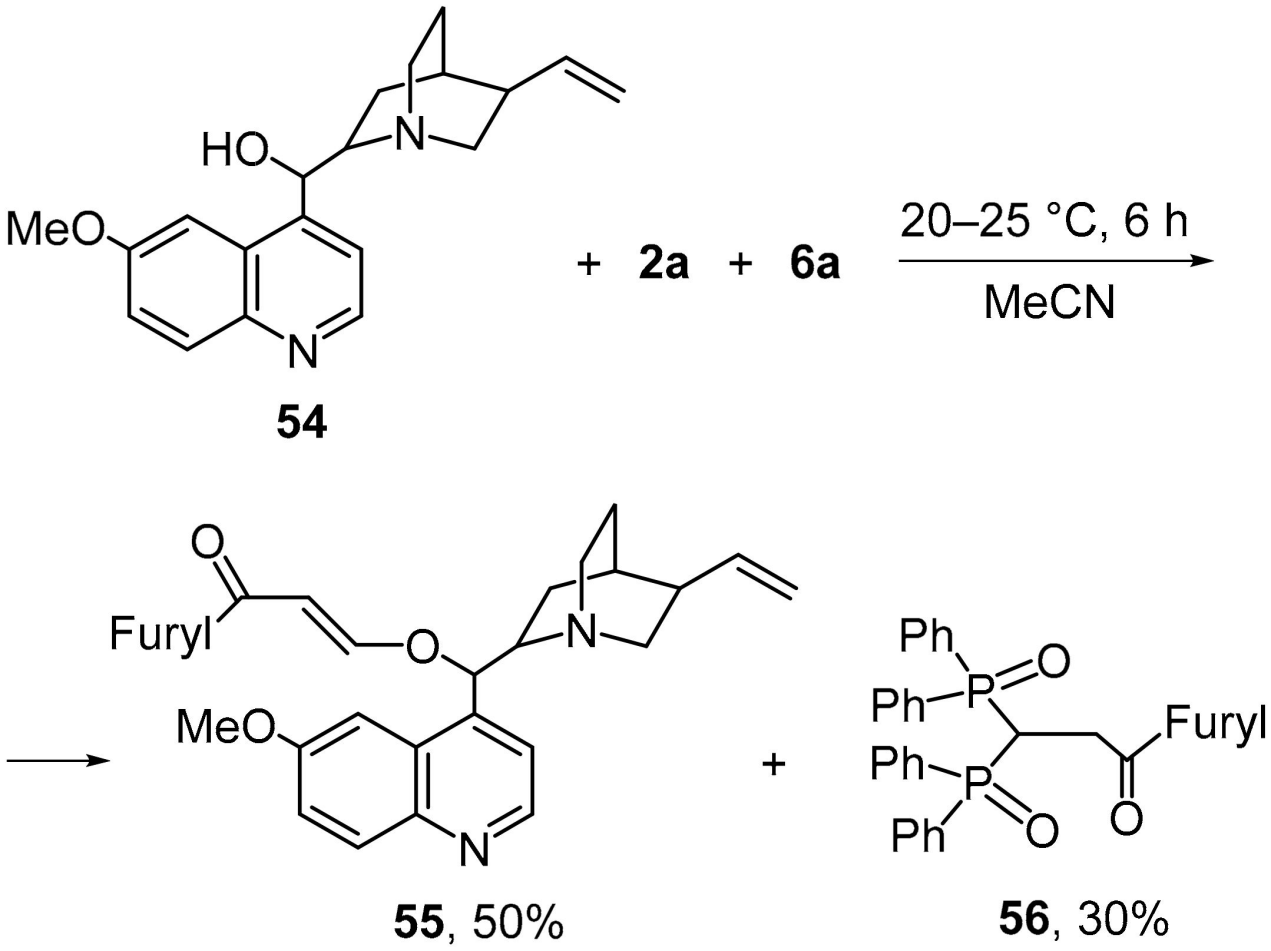
Acylacetylene in the reaction with quinine and diphenylphosphine oxide.

The reaction of quinine **54** with diphenylphosphine oxide **2a** in the presence of furoylacetylene **6a** (20–25 °C, 6 h, MeCN) did not lead to the *C*-phosphorylation of the quinoline core. Instead, the vinylation of the hydroxyl-containing substituent and parallel double addition of the phosphine oxide to the triple bond of acetylene occurred [72] (Scheme 45).

Recently [73,74], it was shown that the promoting role of electron-deficient acetylenes towards the quinoline core in S_N_^H^ reactions can be simulated by the reactive P-H nucleophiles themselves via the reversible protonation of the pyridine nitrogen.

**Scheme 46:**
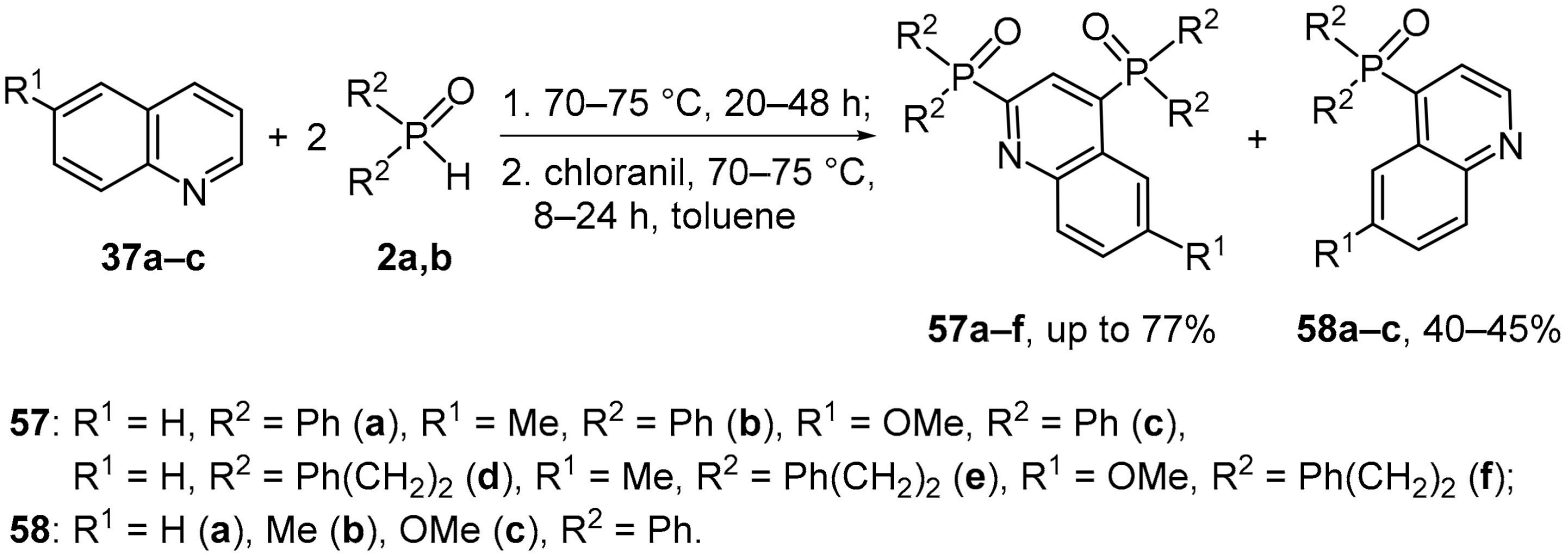
One-pot synthesis of 2,4-phosphorylated quinolines via S_N_^H^ reaction.

Indeed, the reaction of quinolines **37a–c** with secondary phosphine oxides **2a,b** without electron-deficient acetylenes followed by treatment with chloranil [73] led to 2,4-bisphosphorylquinolines **57a–c** along with 4-phosphorylquinolines **58a–c** (for phosphine oxide **2a**) and 2,4-bisphosphorylquinolines **57d–f** (with phosphine oxide **2b**) (Scheme 46).

**Scheme 47:**
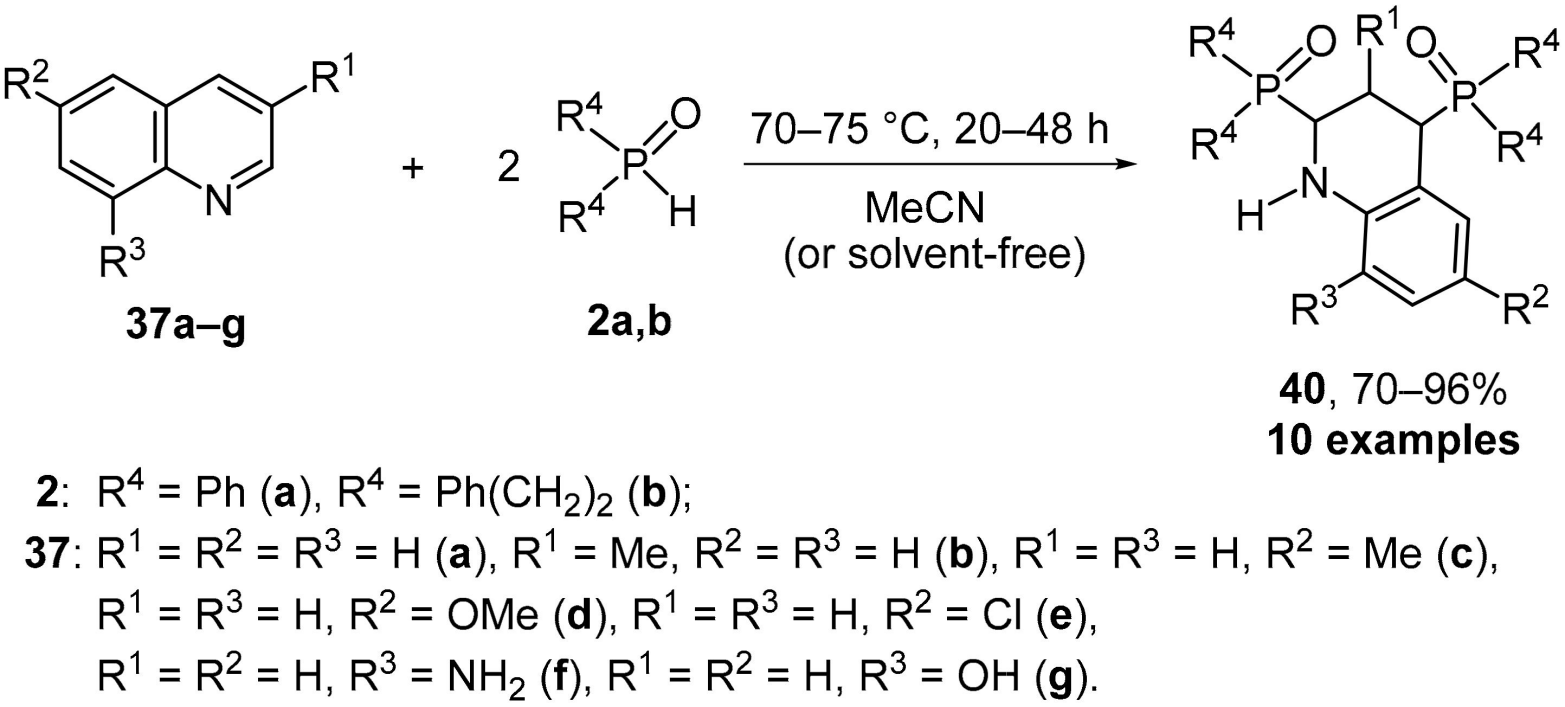
Catalyst-free double addition of secondary phosphine oxides to quinolines.

The intermediates of this unique S_N_^H^ reaction, bisphosphoryltetrahydroquinolines **40**, were observed (NMR) upon the heating of quinolines with phosphine oxides without external oxidants [73]. These intermediates turned out to be stable and isolable in excellent yields (Scheme 47).

The substituents in the quinoline ring slightly affected the reaction time (20–26 h for phosphine oxide **2a** and 46–48 h for phosphine oxide **2b**) and the yields of tetrahydroquinolines **40**, which ranged 81–96% for phosphine oxide **2a** and 70–76% for phosphine oxide **2b**. A lowered reactivity for bis(2-phenylethyl)phosphine oxide **2b** was assumed to be associated with the spatial interference of the voluminous substituent.

The expected monoadducts were not isolated at all, i.e., they were more reactive than the starting quinolines. It was explained [73] by the aromaticity loss of the quinoline core after its first phosphorylation and the increased reactivity of the remaining double bond, which also became more electrophilic due to the effect of the phosphoryl substituent.

**Scheme 48:**
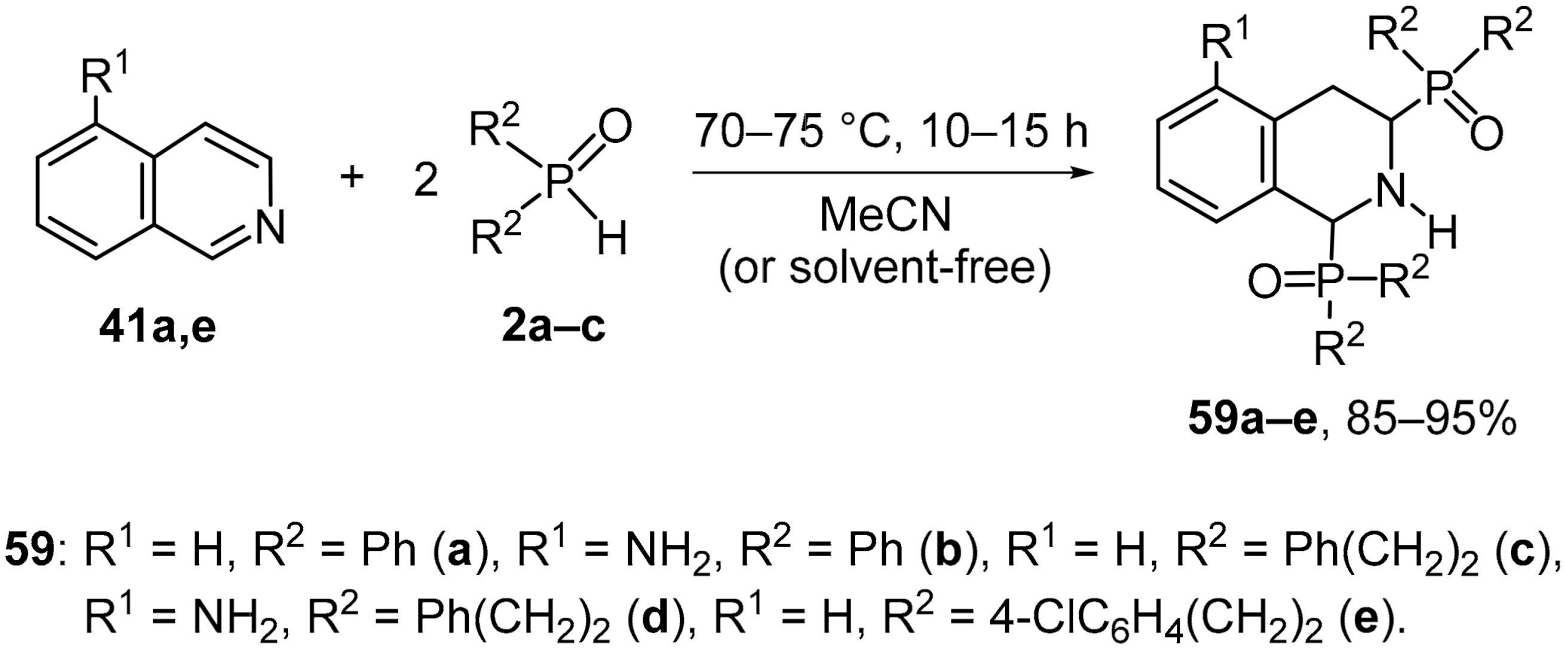
Catalyst-free double addition of secondary phosphine oxides to isoquinolines.

Isoquinolines **41a,e**, when reacted with phosphine oxides **2a–c**, displayed a much higher activity [73,74] as compared to quinolines, also exclusively affording diadducts, 1,3-bisphosphoryltetrahydroisoquinolines **59a–e** in almost quantitative yields (Scheme 48).

**Scheme 49:**
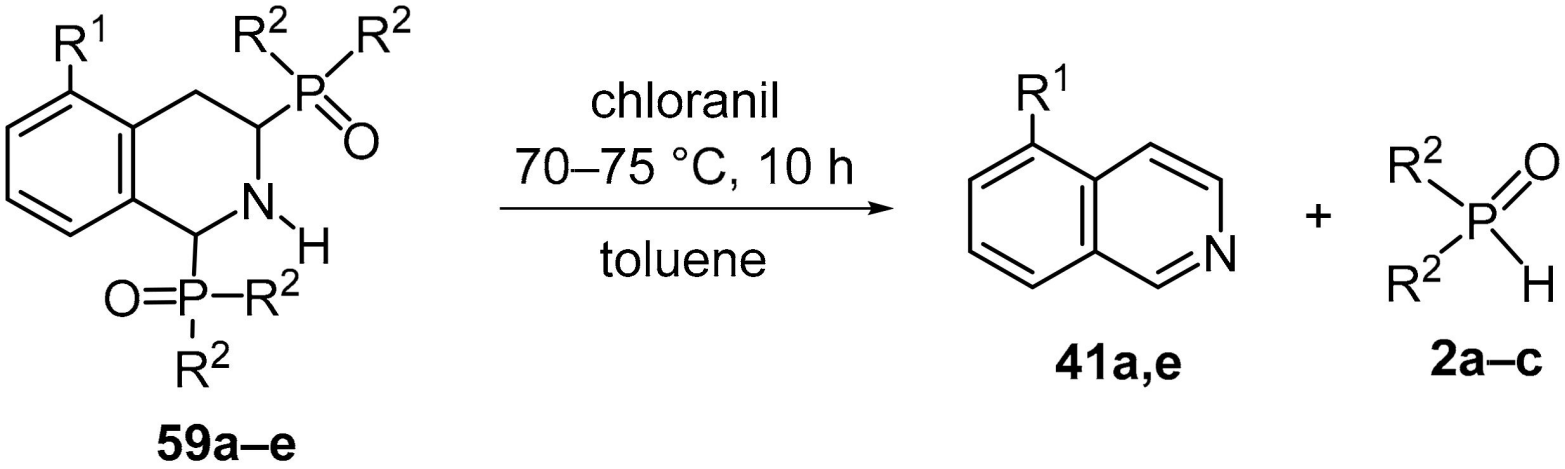
Oxidation of bisphosphoryltetrahydroisoquinolines.

Bisphosphoryltetrahydroisoquinolines **59a–e** when oxidized by chloranil eliminated the starting secondary phosphine oxides **2a–c** to recover the initial isoquinolines **41a**,**e** (Scheme 49) [73].

**Scheme 50:**
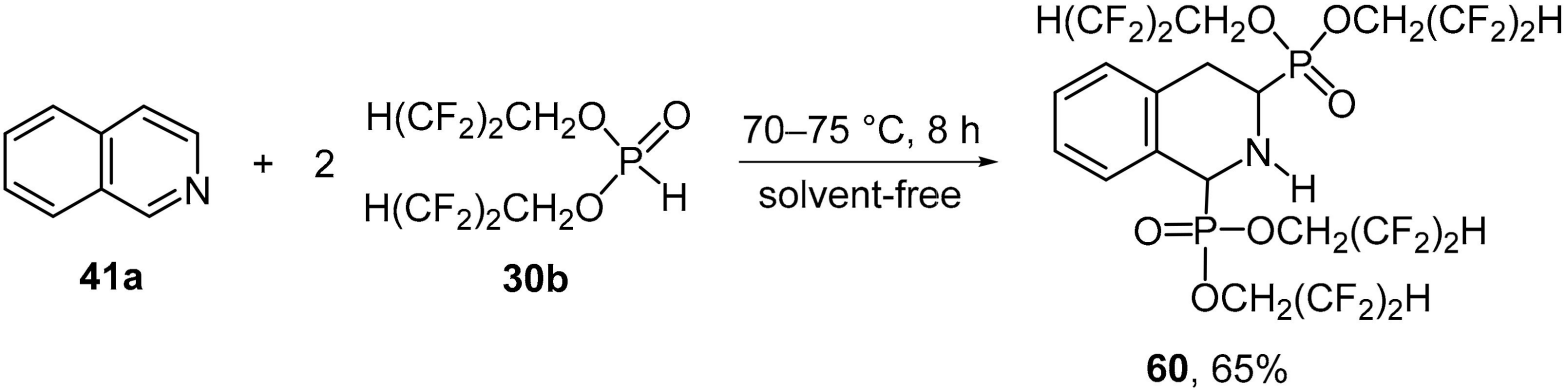
Synthesis of 1,3-bisphosphonyltetrahydroisoquinoline.

The above double phosphonylation of the isoquinoline ring also occurred [73] when *H*-phosphonates, e.g., bis(2,2,3,3-tetrafluoropropyl) phosphonate **30b**, were employed as nucleophiles to afford the expected bisphosphonylated isoquinoline **60** in a 65% yield (Scheme 50).

**Scheme 51:**
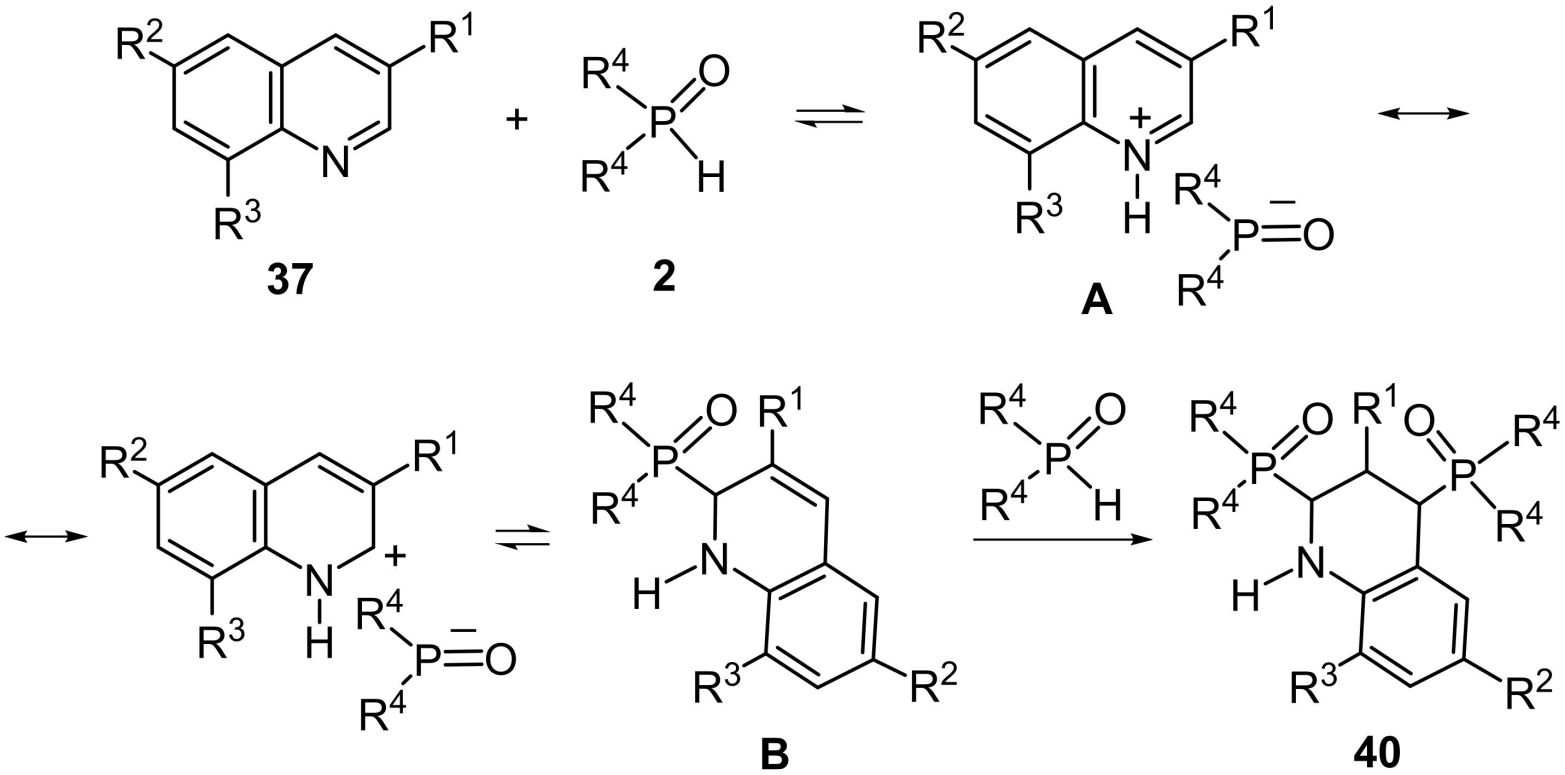
Tentative mechanism of catalyst-free double addition of secondary phosphine oxides to quinolines.

It was assumed [73] that the reaction began (Scheme 51) with the reversible protonation of quinoline **37’s** counterpart by the P-H bond of phosphine oxides **2** to produce ion pair **A** and, next, the monoadduct **B**, which is further phosphorylated to the most stable diadducts **40**.

Diphenylphosphine oxide **2a**, being more acidic compared to bis(2-phenylethyl)phosphine oxide **2b**, deeper protonated quinolines that, in agreement with the above mechanism, facilitated the reaction (Scheme 51).

## 4. S_N_^H^ Phosphorylation of Acridines

**Scheme 52:**
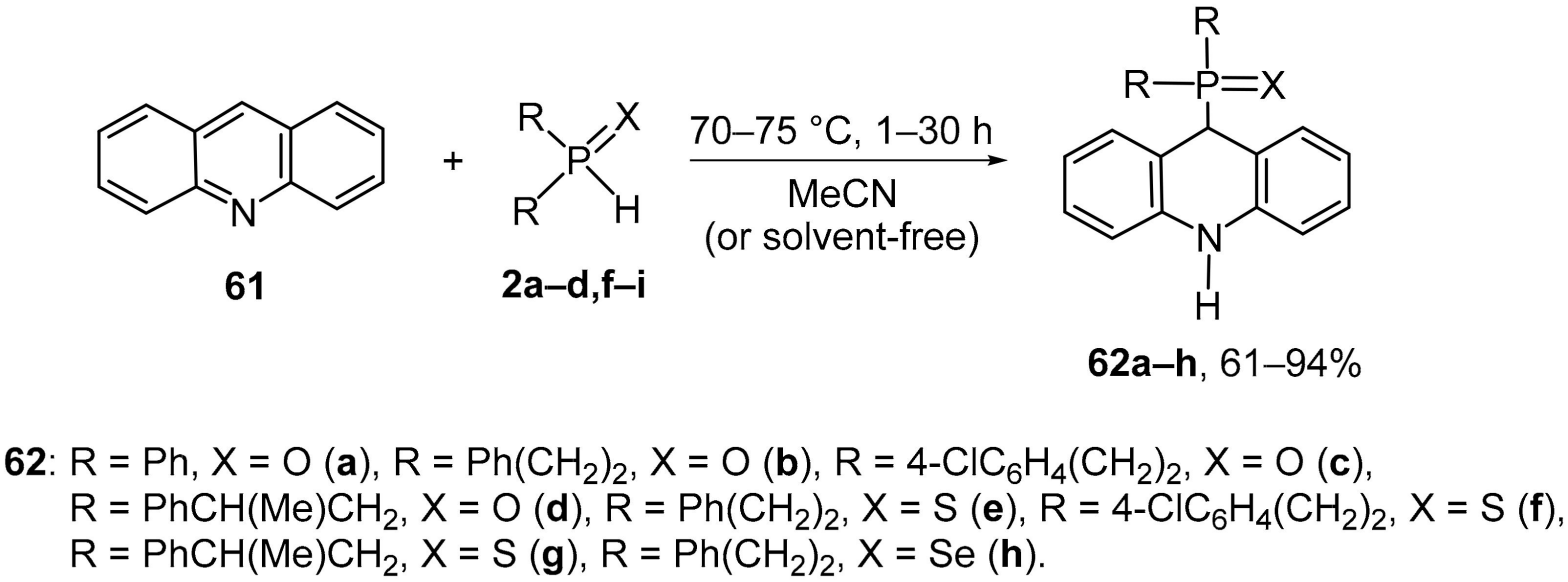
Addition of secondary phosphine chalcogenides to acridine: synthesis of 9-chalcogenophosphoryl-9,10-dihydroacridines.

The attempt to transfer the above-considered (see Section 2.1) cross-coupling of pyridines with secondary phosphine chalcogenides [37] in the presence of electron-deficient acetylenes as adjuvants to acridine series has been undertaken. Unexpectedly, instead of the complete S_N_^H^ reaction, a facile addition of secondary phosphine chalcogenides **2a–d,f–i** to the 9,10-positions of acridine **61** took place to give 9-chalcogenophosphoryl-9,10-dihydroacridines **62a–h** (Scheme 52) [75,76]. Surprisingly, this reaction did not require the presence of electron-deficient acetylenes.

The substituents’ character and the nature of chalcogene in phosphine chalcogenides significantly affect the yields of dihydroacridines and the process duration; selenides appeared to be most reactive, then, followed by sulfides and oxides (Scheme 52, **62h**,**e**,**b**). This allowed suggesting that the proton accelerated the addition process since the selenides were the most acidic [55].

**Scheme 53:**
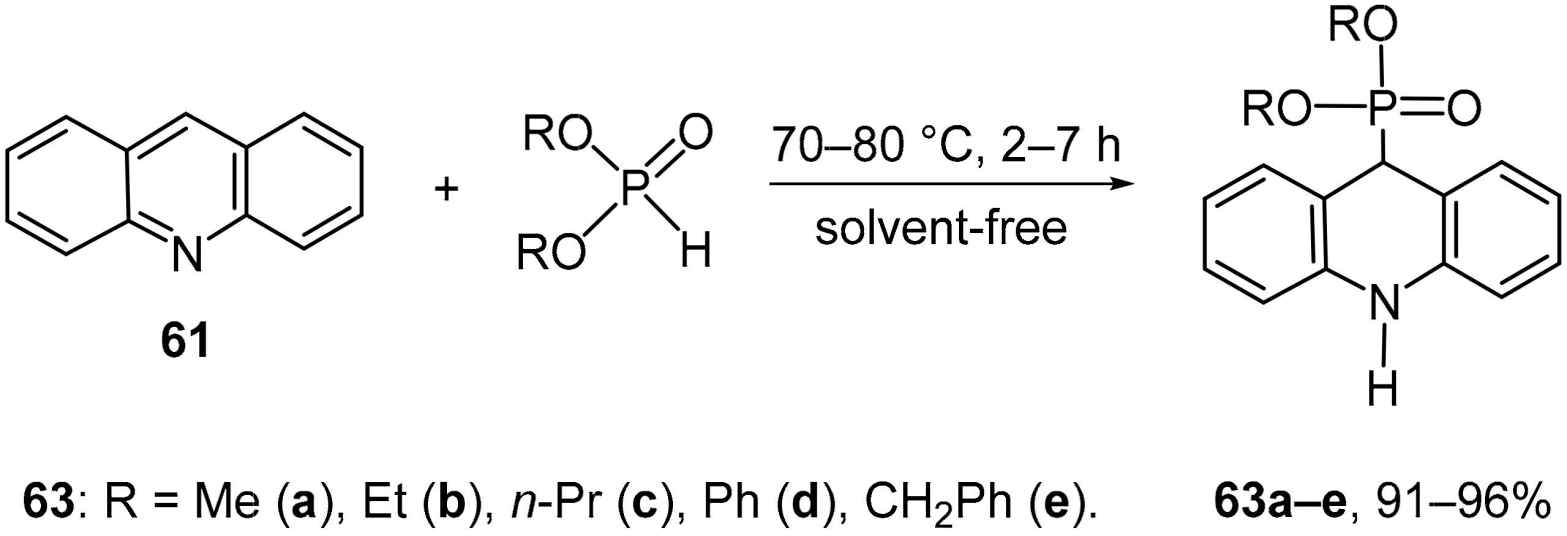
Addition of *H*-phosphonates to acridine.

The reaction was shown to be applicable to dialkyl and diaryl *H*-phosphonates (Scheme 53) [75,77].

Acridine dihydro intermediates were unable to be oxidized (aromatized) by electron-deficient acetylenes such as benzoylphenylacetylene. In the case of dihydroacridines with thiophosphoryl substituents, the restoring of the starting acridine occurred and the eliminated phosphine sulfides added to acylacetylenes [75].

**Scheme 54:**
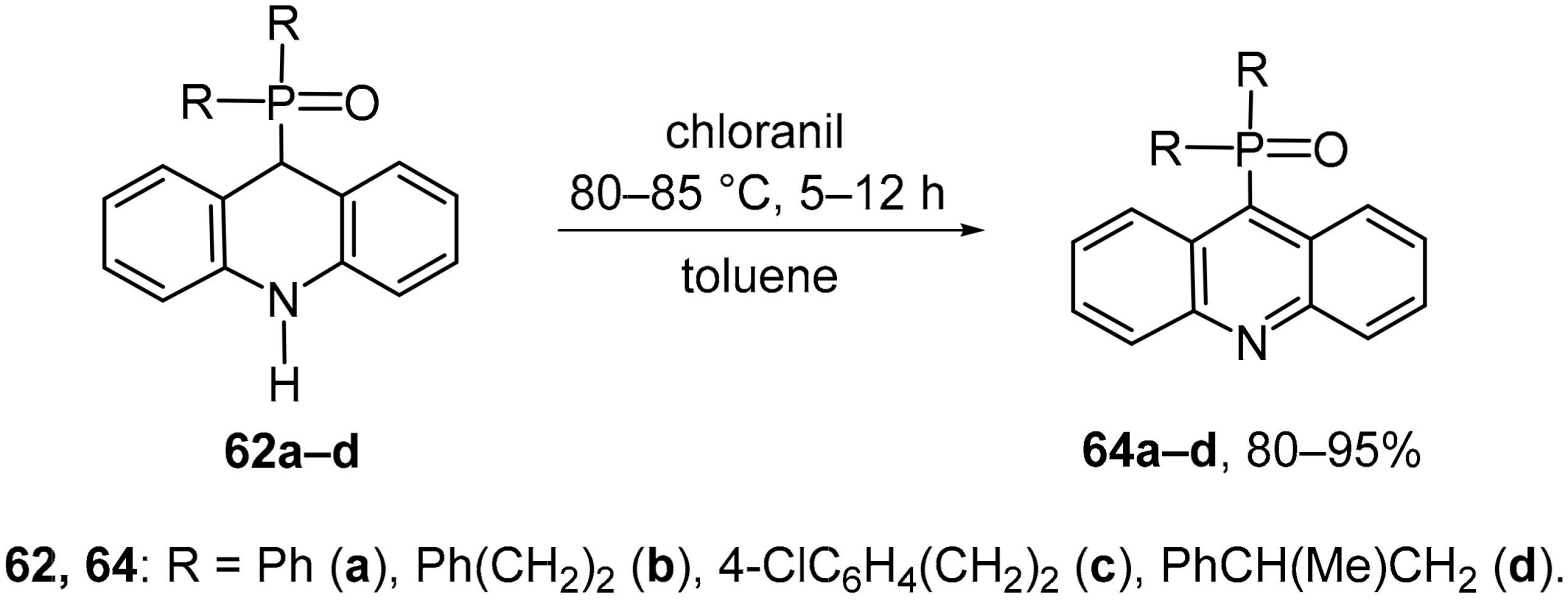
Oxidative aromatization of 9-chalcogenophosphoryl-9,10-dihydroacridines with chloranil.

The anticipated S_N_^H^ reaction was accomplished [75] by the oxidation of dihydroacridines **62a–d** with a common external oxidant such as chloranil. The yields of aromatized products, 9-phosphorylacridines **64a–d** reached 95% (Scheme 54). Tolerable to this reaction appeared to only be phosphoryl derivatives, while sulfur or selenium analogues gave complex mixtures of products.

**Scheme 55:**
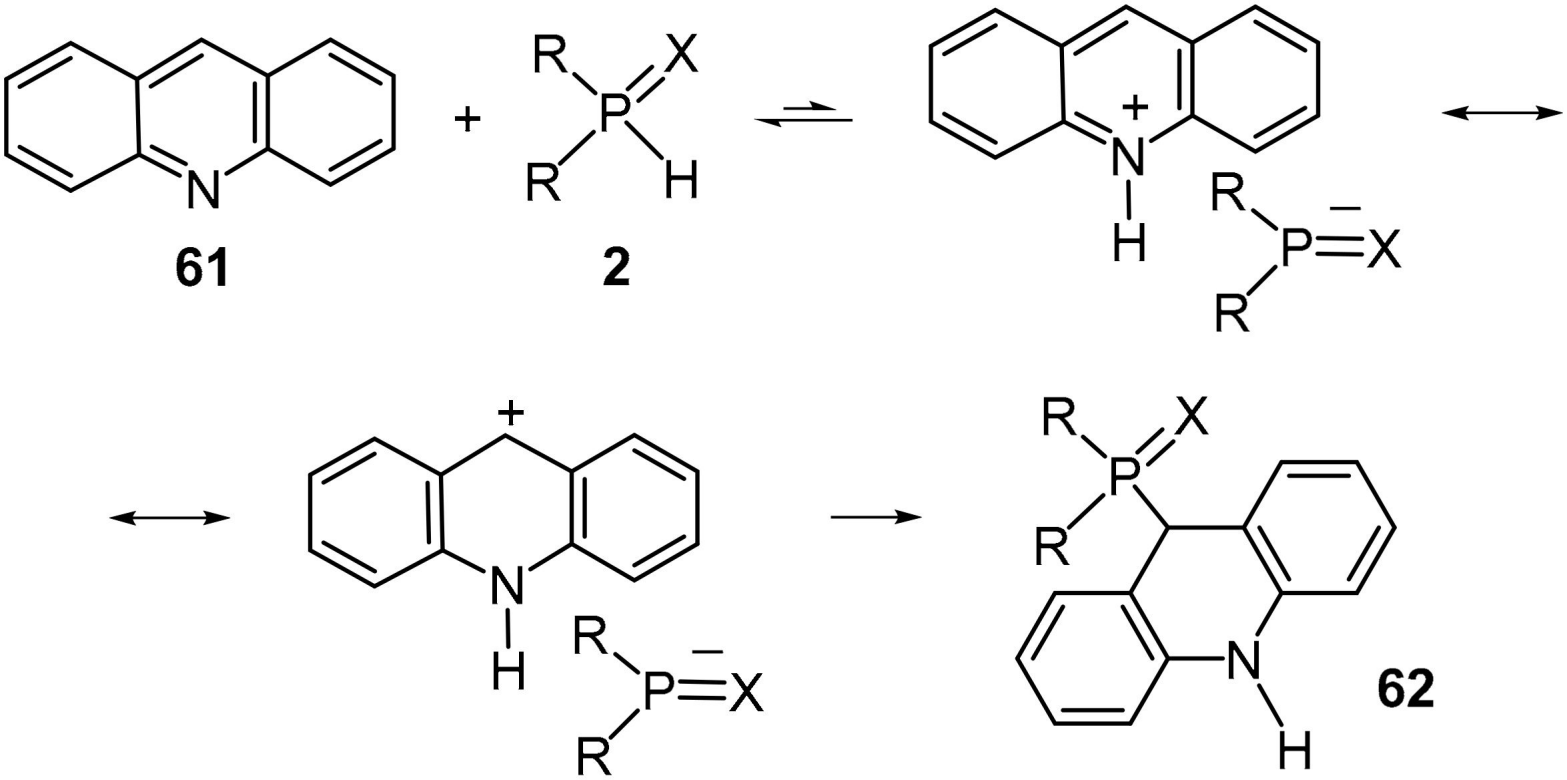
Plausible mechanism of nucleophilic addition.

The resistance of acridine to a one-step S_N_^H^ reaction in the presence of electron-deficient acetylenes was referred to the screening of the nitrogen atom by neighboring benzene ring protons precluding the approach of acetylenes to form the triggering zwitterions. Meanwhile [75], the proton acts as a competitive electrophile and readily attacks the electron lone pair of the acridine nitrogen. The ammonium-like intermediate, thus formed, triggers the addition of phosphine chalcogenides **2** to acridine (Scheme 55). The chalcogenophosphoryl anion, generated by the dissociation of this intermediate, attacks the cationic position nine, to form the final adducts **62**.

The key role of the proton in this mechanism is evidenced by the experiments (Scheme 52) showing that the reaction efficiency (yields and the process duration) improves for more acidic phosphine chalcogenides, as noted above. The carbon analogue of acridine, anthracene, which was not able to be protonated under the above conditions, did not add phosphine sulfide **2f** as checked experimentally. The importance of steric requirements for the attack of secondary phosphine chalcogenides at position nine that follows from the mechanism proposed (Scheme 55) is also confirmed by the experimental results. In fact, higher yields and a shorter reaction time were observed [75] for less voluminous diphenylphosphine chalcogenides and otherwise (Scheme 52).

**Scheme 56:**
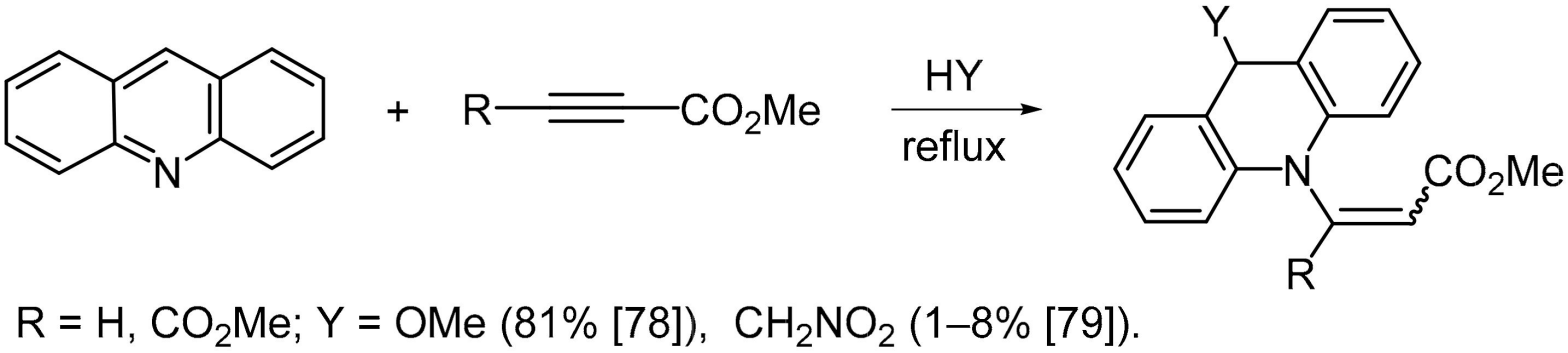
Three-component reaction between acridine, acetylenecarboxylates and methanol or nitromethane.

Remarkably, for the presumably less sterically demanding and more electrophilic acetylenecarboxylates, the nucleophilic attack of acridine nitrogen at the triple bond to generate carbanionic zwitterions is, in some cases, possible, as it was mentioned in earlier publications, wherein the three-component adducts with methanol or nitromethane were formed in 81% [78] and 1–8% [79] yields, respectively (Scheme 56).

Apparently, in these reactions, acetylenes become competitive over protons due to the lower acidity of methanol or nitromethane as compared to phosphine chalcogenides **2** (p*K*_a_ 29.9 and 17.2 for MeOH and MeNO_2_, respectively).

The anodic dehydroaromatization of 9-chalcogenophosphorylsubstituted 9,10-dihydroacridines expectedly proved to be a more efficient and environmentally benign compared to the chemical oxidation.

**Scheme 57:**
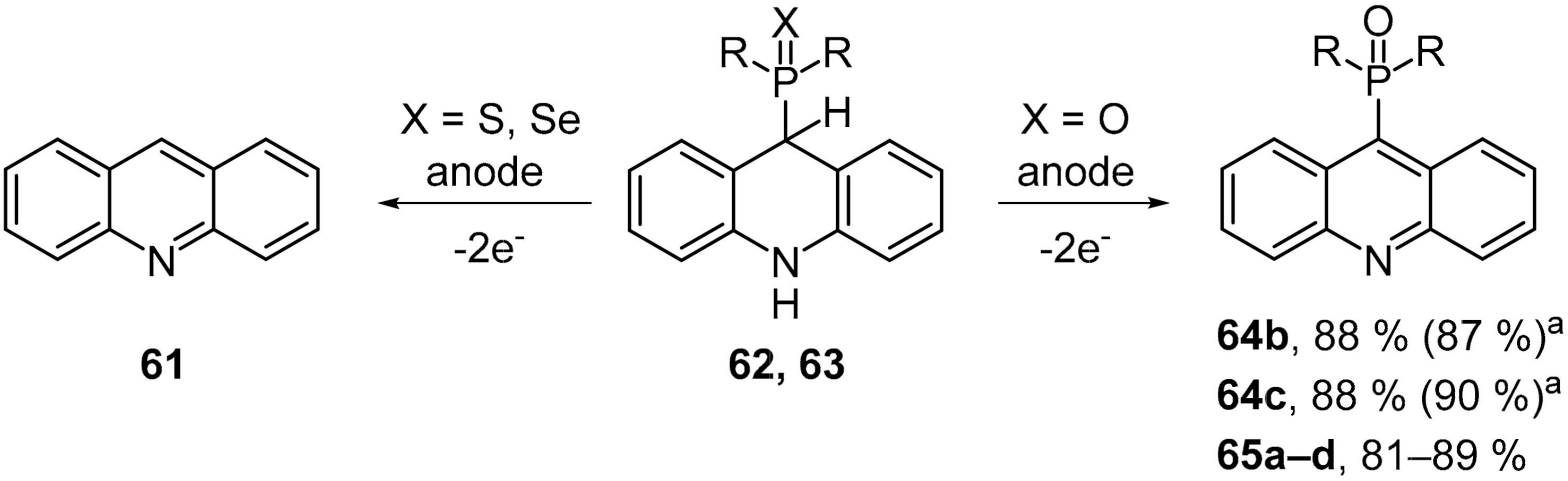
Electrochemical oxidation of dihydroacridines (^a^ Chemical oxidation yields [75]).

Indeed, in the case of dihydroacridines **62, 63**, aromatization proceeded [77] with the saving of the phosphorus-containing substituents to afford the phosphorylated aromatic acridines **64b,c**, **65a–d** in high yields (81–89%) (Scheme 57). However, the decomposition of the starting dihydroacridines with phosphoryl sulfide and selenide substituents **62e,f,h** with the cleavage of the C-P bond was observed (Scheme 57).

The cyclic voltammetry showed [77] differences in the behavior of dihydroacridines **62, 63**. The phosphoryl derivatives **62b,c, 63a,b,d,e** (X = O) gave an irreversible peak of two-electron oxidation, while in the case of compounds **62e,f,h** containing sulfur and selenium (X = S, Se), the voltammogram had two one-electron oxidation peaks. From the quantum chemical calculations of the HOMO followed that the growth of the HOMO population corresponded to a decrease in E_peak_ values [77].

## 5. Conclusions and Outlook

In this survey, the recent data on the nucleophilic substitution of hydrogen in the pyridine nucleus (S_N_^H^ reaction) by PH–nucleophiles (phosphine chalcogenides, *H*-phosphonates) promoted by electron-deficient acetylenes were systematized and generalized. In these S_N_^H^ reactions of a new type, electron-deficient acetylenes play a role of three-modal adjuvants triggering and further driving the whole process. The first modality of such acetylenic adjuvants consists of the activation of the pyridine ring by the reversible formation of 1,3(4)-dipolar donor–acceptor complexes with pyridinoids, which partially transfer their electron density over the anti-bonding orbital of the triple bond so that the repolarization of the system occurs; the former (basic) nucleophilic pyridine moiety now becomes electrophilic, while the acetylenic parts of the complexes acquire a vinyl carbanion character. The second modality is to abstract protons from the P-H bonds to generate *N*-vinyl ammonium-like cations and, correspondingly, *P*-centered anions further recombining to *N*-vinylphosphorylated dihydropyridinoids. The third modality is to aromatize the dihydropyridinoid intermediates by the internal redox elimination of the vinyl moieties, the former acetylenic counterpart, as functionalized alkenes of the *E*-configuration or their oligomers. In a number of cases, this S_N_^H^ phosphorylation of pyridinoids can be retained on the step of dihydro intermediates, usually formed regio- and stereoselectively as 2- or 4-isomers having *E*- or *Z*-configuration of the functionalized vinyl substituents, depending on the structure of pyridinoids and the nature of PH–nucleophiles. From a synthetic and pharmaceutical view, such phosphorylated dihydroazines represent even a higher value than the corresponding aromatized phosphorylated products. It might be expected that this approach to promote the new type of S_N_^H^ reaction can be extendable over other heterocycle/nucleophile/electrophilic acetylene triads. The keys to success of such an extension are (i) significant differences between nucleophilicity of heterocycles and electrophilicity of acetylenes, (ii) the moderate nucleophilicity of nucleophiles not enough for the addition to electrophilic acetylenes (two-component reaction), (iii) nucleophilicity of the anions formed after the abstraction of a proton from its neutral molecules should be appropriate for the addition to the heterocyclic ring activated by acetylenes and (iv) the dihydro intermediates should easily release the acetylenic counterpart (vinyl substituents) to complete the aromatization.

## Data Availability

Not applicable.

## Sample Availability

Samples of the compounds are available from the authors.
